# Supplementary material for: A qualitative inquiry into implementing an electronic health record system (SmartCare) for prevention of mother-to-child transmission data in Zambia: a retrospective study
Source: BMJ Open. 2019 Sep 6;9(9):e030428. doi: 10.1136/bmjopen-2019-030428 (PMC6731929; doi:10.1136/bmjopen-2019-030428)
Supplement: Author's manuscript [file bmjopen-2019-030428.draft_revisions.pdf]

BMJ Open is committed to open peer review. As part of this commitment we make the peer review history of every article we publish publicly available.

When an article is published we post the peer reviewers' comments and the authors' responses online. We also post the versions of the paper that were used during peer review. These are the versions that the peer review comments apply to.

The versions of the paper that follow are the versions that were submitted during the peer review process. They are not the versions of record or the final published versions. They should not be cited or distributed as the published version of this manuscript.

BMJ Open is an open access journal and the full, final, typeset and author-corrected version of record of the manuscript is available on our site with no access controls, subscription charges or pay-per-view fees (http://bmjopen.bmj.com).

If you have any questions on BMJ Open's open peer review process please email info.bmjopen@bmj.com

BMJ Open

A Qualitative Inquiry into Implementing an Electronic Health Record System (SmartCare) for Prevention of Mother-to-Child Transmission data in Zambia

Journal:	*BMJ Open*
Manuscript ID	bmjopen-2019-030428
Article Type:	Research
Date Submitted by the Author:	14-Mar-2019

Complete List of Authors:	Gumede-Moyo, Sehlulekile; London School of Hygiene and Tropical Medicine, Epidemiology and Population Health<br>Todd, Jim; London School of Hygiene and Tropical Medicine Faculty of Epidemiology and Population Health<br>Bond, Virginia; London School of Hygiene and Tropical Medicine Faculty of Epidemiology and Population Health<br>Mee, Paul; London School of Hygiene and Tropical Medicine Faculty of Epidemiology and Population Health<br>Filteau, Suzanne; London School of Hygiene and Tropical Medicine Faculty of Epidemiology and Population Health

Keywords:	PMTCT, routinely collected data, electronic health records

SCHOLARONE™
Manuscripts

A Qualitative Inquiry into Implementing an Electronic Health Record System (SmartCare) for Prevention of Mother-to-Child Transmission data in Zambia

Sehlulekile Gumede-Moyo (BSc, MPH, MBA,)[1,2§]; Jim Todd (MA, MSc)[1] ; Virginia Bond (MSc, PhD)[1,3] Paul Mee (BSc, MSc, PhD)[1] and Suzanne Filteau (BSc, MSc, PhD)[1]

. Department of Population Health, Faculty of Epidemiology and Population Health, London School of Hygiene and Tropical Medicine, London, UK

2. School of Public Health, University of Zambia, Lusaka, Zambia

3. ZAMBART, University of Zambia, School of Medicine

[§]Corresponding author: Sehlulekile Gumede-Moyo. Department of Population Health, Faculty of Epidemiology and Population Health, London School of Hygiene and Tropical Medicine, Keppel Street, London WC1E 7HT, UK. (sehlulekile.gumede@lshtm.ac.uk)

Email addresses of authors:

SGM: sehlulekile.gumede@ lshtm.ac.uk

JT: jim.todd@lshtm.ac.uk

VB: virginia.bond@lshtm.ac.uk

PM: paul.mee@ lshtm.ac.uk

SF: suzanne.filteau@lshtm.ac.uk

For peer review only

Key words: PMTCT, routinely collected data, electronic health records, implementation.

For peer review only - http://bmjopen.bmj.com/site/about/guidelines.xhtml

Abstract

Introduction: Routinely collected clinic data can provide information for surveillance of program implementation and impact. In services for the prevention of mother-to-child transmission (PMTCT) of HIV among pregnant women in Sub-Saharan Africa, the data collection systems often lack immediacy as paper-based records are completed at each facility and aggregate data are collated centrally. Electronic Health Records (EHR) could present an opportunity to improve accuracy, timeliness and scope of surveillance data. This study aimed to investigate the challenges in implementing a Zambian EHR system labelled 'SmartCare' in order to improve PMTCT data.

Method: Data was collected using in-depth interviews, observations and focus groups discussions (FGD) between September and November 2016. Seventeen in-depth interviews were held with a range of key informants from the Ministry of Health and local and international organisations implementing SmartCare. Four data entry observations and three FGDs with 22 pregnant and lactating women seeking PMTCT services were conducted. Data was analysed using a thematic content approach.

Results: The SmartCare system has evolved from various patient tracking systems into a multifunctional system. Funding challenges impede data collection due to manpower constraints and shortages of data collection supplies. Challenges associated with data collection depend on whether a paper- or computer-based system is utilised. There is no uniformity in the data quality verification and submission strategies employed by various implementing partners. There isn't much feedback from the EHR system at health facility level, which has led to disengagement as stakeholders do not see the importance of the system.

Conclusion: SmartCare has structural challenges which can be traced from its development. Funding gaps have resulted in staffing and data collection disparities within implementing partners. The lack of feedback from the system has also led to complacency in operational level which has resulted in poor data quality in later years.

(297 words)

For peer review only - http://bmjopen.bmj.com/site/about/guidelines.xhtml

Strengths

- We obtained a rich and nuanced appreciation of the implementation of the SmartCare as we sampled from a range of IPs, health facilities, stakeholders and implementation systems.
- The depth of the inquiry enabled us to consider a range of explanatory factors.

Weaknesses

- the restriction to health facilities in and around Lusaka

For peer review only

1. Introduction

Routinely collected clinic data can provide much needed information on the prevalence of HIV among pregnant women and the uptake of services for prevention of mother-to-child transmission (PMTCT) of HIV(1). The use of routinely collected data can be timely and cost-efficient for decision making as data are already available for analysis (2). Collection of high-quality routine data on these services and outcomes for HIV-positive mothers and HIV-exposed infants is essential for monitoring and evaluation of PMTCT programmes, for clinical management of patients, and for managing stocks of HIV test kits and drugs (1) For both the clinic staff and health system managers, having access to reliable data that reflects the processes of care and clinical outcomes is the first step to ensuring effective delivery of an intervention within a health care system(3, 4). However in Zambia there is underutilization of routinely collected data in the HIV programmes (5).

In our systematic review of literature of previous work we noted that the data collection systems for PMTCT programs in Africa often lack immediacy as many are paper-based with records completed at each facility, and with individual level data that are inaccessible to central planners(6). Electronic health records (EHRs) could present an opportunity to supplement current sources of routinely collected surveillance data (7). EHRs are real-time, patient-centered records that make information available instantly and securely to authorized users (8). While an EHR does contain the medical and treatment histories of patients, an EHR system is built to go beyond standard clinical data collected in a provider's office and can include a broader view of a patient's care (9).

Various EHR systems are implemented globally (10-12). A systematic of review of literature about the effect of EHR systems in resource-constrained settings recommended that it is urgent to evaluate barriers to implementation (13). Our own research on extracting surveillance data from a Zambian EHR system, SmartCare, has highlighted some deficiencies including large amounts of missing data, especially in more recent years, and variable performance across the country (14, 15). The overall goal of our study is to improve the implementation of an EHR system in Zambia labelled 'SmartCare' and address the fundamental need for timely and high-quality data.

For peer review only

For peer review only - http://bmjopen.bmj.com/site/about/guidelines.xhtml

2. Methods

2.1 Study design

This qualitative study included in-depth interviews, observations and focus groups discussions (FGDs) conducted between September and November 2016.

2.2 Study Setting

SmartCare is a Zambian Ministry of Health (MoH)-led project funded by the United States Centre for Disease Control and Prevention (CDC). SmartCare was developed to improve continuity of care and provide timely data on maternal and child health, HIV/AIDS, tuberculosis and malaria interventions for public health purposes, including Health Management Information System (HMIS) trend reporting and analysis for health officials and clinicians (16).

The SmartCare database is a derivative of the PTS (patient tracking system) which was developed in 2004, based on a health facility-centred EHR system. In 2010, it became a national medical health program and was then rolled out throughout the country. It is implemented by government, and both international and local organisations primarily for patient management. Most of the international organisations were involved in the database development, and the local organisations became involved when implementation was rolled out throughout the country (after 2009).

SmartCare is organised into comprehensive modules and sub-modules ***(Figure 1).*** This was mainly influenced by its funders depending on the information they wanted at that particular time. The main module groups are: clinical group comprising the following modules – ANC, Delivery, Postnatal, ART, Paediatric ART, PMTCT follow up, Under 5 among others – (these are mainly of interest for this research); logistic group which constitute information on drug dispensing and orders; monitoring and evaluation groups which includes health management information system reports, graphing, data analysis, data merge from facilities for MOH; and the continuity of care group which had data from across facilities and within facilities. Through the data merge the SmartCare information can be used to obtain data for the monthly reports to MOH.

Data is captured under a number of 'modules' across a range of health issues. Records are updated at every point of clinical service. SmartCare enables the capture of all patient data

For peer review only - http://bmjopen.bmj.com/site/about/guidelines.xhtml

into one centralised database that can be accessed from any supported health facility. Patients enrolled in the system can have their records accessed at whichever SmartCare-supported facility they present to in the country. Patients are issued with Smartcards at their initial consultation which contains all their clinical information and treatment details. When they present at another facility the health worker simply plugs the Smartcard into the system and can access all the patients' details.

Data on a range of HIV and pregnancy related outcomes are collected during the patient consultation with the clinician, who records the information on paper forms in facilities that are paper-based and directly on the computer in the few facilities which are computer-based. The paper forms are then entered into the SmartCare database by data entry clerks who have been trained in using the SmartCare database. Data is collected from each facility on a monthly basis and submitted to a District Health Information Officer, who aggregates and sends the data to the province level Senior Health Information Officers. From here the data are wired to a national server at MoH headquarters ***(Figure 3)***

For the purposes of this study, routine data is defined as data that are routinely generated through antenatal care (ANC) and PMTCT service delivery, and routinely recorded in standard SmartCare data tools.

2.3 Sampling

The implementing partners (IPs) were purposively sampled in consultation with the MoH to identify diverse organizations. These included both well-resourced international organizations and less well-resourced local and government-owned organizations. Within each of the selected IPs, a diverse range of interviewees were purposively recruited in order to give the broadest range of perspectives (17, 18). The approaches to recruitment of participants were flexible, being negotiated with key contacts and recommendations from managers who were also considered for in-depth interviews. The FGDs were conducted at maternal and child health (MCH) departments in health facilities among consenting pregnant and lactating HIV-positive women on ART.

Six health facilities from the representative IPs were also purposively selected based on local considerations which included, among these facilities: those best performing versus struggling; those which are paper- or computer-based; and facilities which had already transitioned to 'test and treat' for general population HIV care. Most of the facilities sampled

For peer review only - http://bmjopen.bmj.com/site/about/guidelines.xhtml

were in Lusaka City, and only one was peri-urban. The choice of Lusaka was based on the following considerations: 1) logistics, 2) facilities in Lusaka are busy and have the highest case loads, 3) our previous work has shown that facilities in Lusaka show a wide range of successes and failures, and 4) overall Lusaka performs poorly compared to the rest of the country and therefore it was important to investigate the working of SmartCare in Lusaka city.

2.4 Data sources

Data were generated from 17 in-depth semi-structured interviews, four data entry observations and three FGDs with 22 pregnant and lactating women seeking PMTCT services from three health facilities (Table 1). The face-to-face semi-structured interviews were held with a range of key informants from MOH, and local and international organisations SmartCare IPs. IP informants were from leadership, operational and implementation levels (Figure 1). The key informants for implementation levels were based in the 6 sampled health facilities, whilst in-depth interviews with leadership and implementation level participants were carried out in their work places. For leadership staff and some senior operational level stuff the interviews were carried out in their individual offices. For the operational level participants who were in shared office space, interviews were carried in boardrooms.

Interviews with key informants were conducted by the first author in English. They lasted 30 to 60 minutes and consisted of a series of open-ended questions with follow-up question probes. The interview guides were specific to the hierarchical levels of operation, namely: leadership level (persons involved in the strategic direction of SmartCare); operational level (Data Managers and Monitoring and Evaluation personnel); and implementation level (Data entry clerks and MCH in-charge clinicians). The interview guides were designed to capture information on SmartCare development, implementation procedures, data quality management and linkage between SmartCare and PMTCT services. The FGD sessions were conducted by the first author and a co-moderator who was fluent in the local languages (Bemba and Nyanja). These discussions were guided by an interview guide containing open-ended questions and lasted for 60 to 90 minutes. The interview guide was designed to capture the general knowledge and experience of the PMTCT programme and SmartCare operations Two FGDs were carried out in different Lusaka health facilities, and one in the peri-urban area.

For peer review only

For peer review only - http://bmjopen.bmj.com/site/about/guidelines.xhtml

The observations were conducted to familiarise the researcher with the realities of the SmartCare data collection and entry environments. These were planned to be conducted in 6 health facilities through interaction with study participants and relevant actors as well as personal reactions to the related events without the use of a specific tool. However in 2 health facilities data entry was not taking place on the scheduled visit days due to power outage, hence only in-depth interviews were conducted with implementing level stuff only.

2.5 Data Management and Analysis

The in-depth interviews and FGDs were tape-recorded and subsequently transcribed verbatim. FGDs were then translated into English. Data was collected aiming to get sufficient data from relevant actors/perspectives to ensure it was qualitatively representative (19). Detailed field notes were taken on the sites following the observations for analysis.

Data were collected and analysed without a constraining overarching framework, which enabled us to capture the diversity of experiences of the SmartCare implementation efforts. A thematic content approach was conducted; this was done by reading the transcribed material systematically, line by line, in order to identify the meaning units. Meaning units were defined as strings of the text expressing a single coherent thought, up to the point at which the coherent thought changed. Thereafter, the meaning units were marked by a code, describing what the text unit was about. Coding was also done without any pre- conceived ideas.

The codes were defined and organised so that those referring to the same subject were grouped into categories. The interview guide was used as a starting point for grouping the information, but during the analysis new categories were developed. When this happened, we re-coded. The data was managed manually in Excel sheets. The underlying meanings of the categories were formulated into a theme. The original data was re-assessed by an assistant (MSc Student) after analysis in order to detect any concepts or information that would have been missed. The first author discussed the results with SF, JT and the MSc student.

3. Research ethics

Written informed consent was obtained from all in-depth interviews participants including their consent to record the interviews and publish anonymous quotations. Verbal consent was sought for FGD participants. Participation in the study was entirely voluntary and participant anonymity was maintained throughout the processes of interview transcription, data analysis and presentation by using pseudonyms. Permission to collect data from the health facilities

For peer review only - http://bmjopen.bmj.com/site/about/guidelines.xhtml

was granted by the MoH district offices. The study was approved by the Zambia Biomedical Ethical Board (Ref 101-04-16) and the LSHTM Research Ethics Committee (Ref 12086).

4. Results

The comments from key informants, observations and the focus group discussions enabled us to characterise four fundamental issues that are key in the implementation of SmartCare: database development and ownership, funding and staffing, the data collection process, and health facility set up.

4.1 Database Development and Ownership

The development of SmartCare was mainly CDC-led, with the government of Zambia through the MoH supporting the process. Multiple stakeholders using various patient tracking software were brought together to form SmartCare. Reflecting on the history of SmartCare, two IP informants identified the leading role of CDC and the subsequent collaboration with a broader group of partners.

'The initial development was by the American-funded institutions and a few people from the MoH because they had gone into partnership. They were developers at MoH and also at CDC, the rest of the institutions like us were only required to use the software.'- Implementing partner leader.

'There were other systems that were used by other partners throughout the country e.g., there was a system called the CareWare which was similar to SmartCare but it was just a patient summary. Then there was another system which was used in the private sector which l can't remember the name. Lessons were drawn from all these systems and brought into one and this is how SmartCare was born.'- Implementing partner leader.

There was a consensus among the leaders of the SmartCare implementation that the database has also evolved from a system to track patients to its current form as a mixture of a patient tracking tool, a clinical care tool, a reporting tool, and a surveillance tool. This was as a result of different stakeholders who had different data needs which took the form of added modules. The changes have however, made it difficult to classify and use the database because it now tries to address multiple issues simultaneously.

For peer review only - http://bmjopen.bmj.com/site/about/guidelines.xhtml

*'I wanted my things as well and partly l wanted things that PEPFAR (*The United States President's Emergency Plan for AIDS Relief) *wanted because our money comes from them. If I can't report on PEPFAR indicators, l don't get more money' –Implementing partner leader.*

'...it was just a mixture of ideas which were clinically required but each person had different needs for information, some needed it for research purposes, l needed it for patient follow up, another person needed it for forecasting and quantification'- Implementing partner leader

The resulting SmartCare forms are 6-7 pages for a normal interaction per visit because of requirements from its various stakeholders. In addition, a lot of data has been entered into the system making it bulky. This has caused the system to become very slow, and in the mornings it takes a very long time to reboot as narrated by operational and implementing level participants. Data entry is often interrupted as the system also hangs up because it is so bulky. Hence a lot of backlogs are experienced which results in a lot of data not being entered.

'Because the system is slow they have to wait instead of entering 100 files per day they are only able to enter 60 or so.'- Database Manager.

'Last year it had serous faults and we were not using it for a while' – Data Entry Clerk.

Although there is a SmartCare technical working group, chaired by the MoH where operations are discussed, it is not clear who is guiding the process, and what should be added into the database to make it work better.

4.2 Funding and staffing

The database operations are managed by the monitoring and evaluation (M&E) departments of the IPs. The structures of the M&E departments differ across the IPs, with international organisations having much bigger and better resourced departments. The local IPs have much leaner structure, rely mostly on volunteers, and run into problems due to financial constraints. This was elaborated by the leadership and implementing level participants.

'The positions of data entry clerks in the facilities are actually paid for by the donor. It's not sustainable because the moment the donor says we are pulling out, there will not be any data entry personnel in these facilities.'- Implementing partner leader.

For peer review only - http://bmjopen.bmj.com/site/about/guidelines.xhtml

Mostly the people who enter data in the health facilities where we operate are employed by us. Apart from that we also provide support through volunteers; in most cases we also provide peers and counsellors. –Data Base Manager.

The manpower constraint in the local partners has resulted in the loss of data during long grant negotiations. Data is lost as data entry staff are laid off between funding cycles since their salaries are dependent on grants. In addition, these partners have designated personnel who enter general ART data whilst PMTCT data is mainly entered when the data entry personnel have specific interest or have trained temporary volunteer staff.

The funding of operations also affects the supplies required for the day to day operations as narrated by both operations and implementing personnel.

'We normally run out of forms, especially when funding is out. When we don't have funding the facilities also have challenges in printing the forms because toner is expensive.'- Data Entry Clerk.

'....when the computers are down because of the virus, data entry doesn't happen.'- M &E Officer.

In some small facilities where the MoH is solely responsible for data collection and entry, there are no data entry clerks and hence clinicians enter the data on the computer after hours. This is usually done only by staff that are passionate and so give it time according to a MoH leader.

'In the government facilities there is no designated person to manage ART data for SmartCare; its health workers who just do that on their own. - 'Implementing partner leader.'

For peer review only

4.3 Data Collection Process

.4.3.1 Data entry

The SmartCare data is collected either directly onto a computer or using a paper-based method. When using the computer-based data collection and entry method, clinicians have to enter data directly in the computer; this method is still being piloted in a few facilities mainly in the Southern and Lusaka provinces. For the paper-based data method, client information is documented in the SmartCare care forms. The papers are then taken to the data entry room where the information is entered into the computer. In some facilities which are deep rural

For peer review only - http://bmjopen.bmj.com/site/about/guidelines.xhtml

(where there is no electricity at all) they are using paper only and the forms are then transferred to a location where there are computers and entered into the database.

With both the computer and the paper-based methods the greatest challenge is incomplete forms which translate to missing data in the database. In some facilities which are computer-based, the data entry clerks noticed that there are also some clinicians who still prefer entering data on paper and handing over to the data entry clerks to enter on the computer.

'In as much as data is supposed to be entered at each service point, some people prefer entering the data on paper and they hand over the data to me.'- Data Entry Clerk

According to implementing level staff, with the paper-based method, once the client leaves the facility it is a challenge to follow up on the missing data on the forms.

'There are usually many gaps in the forms; the forms are not fully completed more than half of the time.'- Data Entry Clerk.

'When the forms are not complete l return them to whoever was supposed to be entering the data at that service point but the challenge is that they will tell you the person has gone and that can't follow them up and even so in the next visit they will not even bother correcting the information'- Data Entry Clerk.

One of the MoH participants explained that the incomplete data forms could be a result of the competing demands to physically attend to clients and fill out the forms in the high volume facilities.

'.... It takes 20-30 minutes just to enter the data for one interaction. In clinics where there are many patients, how many patients are you going to see in a day?' – Implementing partner leader.

The implementation challenges of the SmartCare database depend on the data collection and entry method used. Power outages were the greatest challenge: under the computer-based method, client information is not collected, as some clinicians will be reluctant to use the alternative method, whereas with the paper-based method there will be a backlog of work.

Some well-funded partners have installed solar power systems in the health facilities, and this enables them to enter data without interruptions. Despite having good power supply, there is

For peer review only - http://bmjopen.bmj.com/site/about/guidelines.xhtml

a challenge in these facilities of some clinicians who are not computer literate. Furthermore, because of other competing health facility priorities, the power is rarely used for data entry.

'......would you want to power theatre activities or you want to power computers for data entry? Theatre is life saving and data can be entered anytime.' – Implementing partner leader.

4.3.2 Data quality verification

Verification of data quality is specific to each implementing partner. The verification process involves comparing the patient information on the paper forms and registers against the information in the database. The international partners also have verification procedures imbedded in the Monitoring and Evaluation strategies as narrated by one of their participants.

'We are doing double data entry for 10% of our files.' – Data Manager.

On the other hand, verification is rarely done by the local organisation due to manpower constraints as indicated by an operational level participant.

'When I get time, l check what is in the system versus what is in the registers. However it's rare that l get that time because l always have files to enter into the system. In that case l wait for the Quality Control people from Head Office to come and do random verifications for me.'- Data entry clerk

All the partners reported that there are some instances where they can't find patient files, making it difficult for them to verify the data that is in the computer with the raw data.

'There are certain instances where we can't find patient files'. – M&E Officer.

The patient files will tell me that a facility has 200 clients whilst SmartCare has 300 clients; this is because some of these files are kept somewhere, could be files of staff members or people with prominent positions in that particular area'. – Database Manager

The SmartCare Database system has reports that are built in the application. However all the partners alluded that these reports are very inconsistent and incorrect.

'In some cases it will show a large number of people who would have been initiated in a day more than what we would have actually done.'– MCH Nurse in Charge.

For peer review only - http://bmjopen.bmj.com/site/about/guidelines.xhtml

4.3.3. Submission of data to the main database

There is also no uniformity in submission of the data to the main database, although according to the MOH the data is supposed to flow from the facility to the district, province and then to the main database at head office (Figure 2). Some partners wait for MoH to request data whilst some submit their data to CDC.

'They sometimes come to the facilities and collect the data on their own since they are the ones who own these facilities as well' – M & E officer

'We do not follow that procedure as per say because we collect data directly from the sites and aggregate it from here and submit to CDC. If MOH requests it, we submit to them but we are not that consistent'- Database Manager

4.3.4Feedback from Database

There is no standardised procedure for reporting data that is collected by IPs. The partners normally use the information internally as well as sharing with the donors supporting them.

'We also have special reports that we do in a monthly basis such as the PEPFAR report' – M & E Coordinator.

In contrast, the nurses-in-charge in some of the facilities reported that they have never received feedback from the system.

'We have not received anything; they don't give us any reports. We would, however, want to receive reports so that we would know how we are fairing.' – MCH Nurse in Charge.

As viewed by one of the leaders who participated in the study, the lack of feedback from the system has led to disengagement and some stakeholders not seeing the importance of documenting client information in the SmartCare Database.

'*A lot of the staff collecting this data do not understand how and why they are collecting this data and they are frustrated by it as it also takes lots of their time entering this data' – Implementing partner leader.*

4.4 Health Facility setup

The set up in some health facilities is in such a way that the PMTCT services are offered in the ANC department whereas ART services are offered in places designated solely for ART.

For peer review only - http://bmjopen.bmj.com/site/about/guidelines.xhtml

For peer review only

The nurses in charge in such health facilities narrated that the facility setup could be a great challenge in ensuring that the PMTCT data is entered in an efficient manner.

'Probably if it was housed within the ART department, you could have the same person entering the PMTCT data into the SmartCare' – MCH nurse in charge.

In addition, the MCH nurses-in-charge indicated that client files sometimes go missing between the MCH and the data room.

'I am sure when they are sending the files; some are lost during transfer in different departments.' – MCH nurse-in-charge.

Overall, women seeking PMTCT services did not have concerns on the implementation of SmartCare, as according to them the healthcare workers will be doing their job.

'The person who is supposed to enter the data does so because he was trained anyway'- PMTCT FGD Mothers.

'We just carry our cards and leave them to do their job'- PMTCT FGD Mothers.

'We are here for our lives and that of our babies, we have to comply and be patient'- PMTCT FGD Mothers

5. Discussion

The study has provided evidence on the SmartCare implementation challenges. It has also provided insights as to how to improve implementation, and data quality. The main points were to collect less, but better data, to engage the clinic staff by providing regular feedback, and to improve the software. Fortunately, PMTCT clients appear already satisfied with the system.

The design of EMR systems does not always get attention due to pressures related to their functionality requirements(10). SmartCare was exposed to pressures as its data model evolved from a patient tracking system into a multi-functional system due to demands of various stakeholders. In an effort to shape the SmartCare database into a manageable tool that can be used for public health purposes, CDC and MOH could streamline the process for including and excluding information that is collected. There is a need to cut down on the number of fields collected so that less data of high-quality data is collected. This could

For peer review only - http://bmjopen.bmj.com/site/about/guidelines.xhtml

improve the system by: reducing the amount of data so that it takes less time to collect and people might more readily do it; and having a less bulky database which will be less likely to be affected by computer crashes and faster to use.

SmartCare could be developed into an Open Medical Record System (MRS), which has been reported to be successful in other developing countries like Rwanda, and Kenya (20). The system could also be upgraded such that: data can be accessible and be shared in multiple sites; multiple users can enter data simultaneously; data can be backed up automatically at more than one site; information can be communicated between multiple locations; data are accessible and shared at multiple sites and the system can be debugged and upgraded over the internet without visiting remote sites (10, 21).

In Ethiopia the quality of data was reported to be affected by dual documentation where both paper based and electronic systems were used (22). Learning from the Ethiopian experience, it is important that there is uniformity in the implementation of SmartCare throughout the country so that everyone will be on the same level. This will allow for comparisons across geographic regions and longitudinal analyses.

In our findings workload affected the documentation, as evidenced by poor data quality in the quantitative analysis of SmartCare PMTCT data (14, 15). In low –resource countries there is a shortage of a qualified work- force and most health care institutions do not have dedicated IT staff for their EMR systems (23). The results of our study are consistent with a systematic review of literature on the role of EHR systems in developing countries which pointed to the lack of financial and human resources as major challenges in implementing EMR systems (24). Therefore, there is a great need for strong commitment from both the government and the donors to invest in improving the implementation of the system.

There was a notable lack of appreciation of the system, and a need to train and support end users of the system such as the clinicians who are directly involved in the data collection process. For both the clinic staff and health system managers, having access to reliable data that reflects the processes of care and clinical outcomes is the first step to ensuring effective delivery of an intervention within a health care system(4). Hence, there should be better buy-in of the staff at the facilities in order to make sure that they fill in the forms regularly and accurately. The data from the database should be presented to facility staff as an advocacy tool which is needed in their facilities, so that they would appreciate the need for it (25, 26).

For peer review only - http://bmjopen.bmj.com/site/about/guidelines.xhtml

Similarly, in their description of the rationale and experience in scaling up EMR in Malawi, Douglas and colleagues concluded that health workers will not adopt a system if they do not find sufficient value in it (27).

Strengths and Limitations of the study

We obtained a rich and nuanced appreciation of the implementation of the SmartCare as we sampled from a range of IPs, health facilities, stakeholders and implementation systems. The depth of the inquiry enabled us to consider a range of explanatory factors. Most of these factors were related to the EHR system and its implementation. We also considered the factors encountered by the PMTCT clients but these did not emerge as major concerns.

There are several limitations of the study, such as the restriction to health facilities in and around Lusaka. Lusaka is the capital city of Zambia and therefore the health facilities selected could be receiving financial and technical support of an order that is unlikely to be offered in remote facilities even by the same IP. However, our previous work on the quantitative analysis of PMTCT data does not support this argument as several provinces had better data quality than Lusaka (14, 15).

6. Conclusion

The SmartCare system has structural challenges which can be traced from its development. Funding gaps have resulted in staffing and data collection disparities within implementing partners. The lack of feedback from the system has also led to complacency in operational level which has resulted in poor data quality in later years. The data from the database, if appropriately understood, could be used by health facility staff as an advocacy tool, as well as in monitoring the impact of PMTCT programs. Our research could aid other countries wanting to develop their own EHR systems.

(4520 words)

Summary

What is known

- Electronic Health Records (EHR) could present an opportunity to improve accuracy, timeliness and scope of surveillance data

For peer review only

- Data collection systems for PMTCT programs in Africa often lack immediacy as many are paper-based with records completed at each facility, and with individual level data that are inaccessible to central planners.

What this study adds on

- There is need to invest in EHR systems for them to be implemented in a very efficient manner.
- It is important that there is uniformity in the implementation of EHR.
- Feedback from the system must be presented to stakeholders.

Authors' contributions

SGM conceived the study and conducted all the interviews and observations under the supervision of SF, and JT. Analysis was conducted by SGM with the input from SF, JT and VB. The manuscript was drafted by SGM with contributions from all the authors. All authors read and approved the final manuscript.

Availability of Data

No additional data available.

Competing interests

None declared.

Acknowledgements

The study was supported by the SEARCH (Sustainable Evaluation through Analysis of Routinely Collected HIV data) Project funded by the Bill & Melinda Gates Foundation grant number OPP1084472. We also thank the Ministry of Health, Department of Policy and Planning for granting us permission to access the SmartCare database. The authors thank all the participants who contributed their time and effort to the study and Jasleen Singh (MSc student) for going through the transcripts and assessing the initial analysis.

For peer review only - http://bmjopen.bmj.com/site/about/guidelines.xhtml

References

1. Gourlay A, Wringe A, Todd J, Michael D, Reniers G, Urassa M, et al. Challenges with routine data sources for PMTCT programme monitoring in East Africa: insights from Tanzania. Global health action. 2015;8:29987.
2. Grzeskowiak LE, Gilbert AL, Morrison JL. Methodological challenges in using routinely collected health data to investigate long-term effects of medication use during pregnancy. Ther Adv Drug Saf. 2013;4(1):27-37.
3. Shaw V. Health information system reform in South Africa: developing an essential data set. . Bulletin of the World Health Organization. 2005;83(8):632–6.
4. Mate KS, Bennett B, Mphatswe W, Barker P, Rollins N. Challenges for routine health system data management in a large public programme to prevent mother-to-child HIV transmission in South Africa. PloS one. 2009;4(5):e5483.
5. Munthali T. MP, Mee P., Gumede S., Schaap A, Mwinga A., Phiri C., Kapata N., Michelo C., and Todd J. Underutilisation of routinely collected data in the HIV programme in Zambia: A review of quantitatively analysed peer-reviewed articles. BMC Research Policy and Systems. 2017;15(51).
6. Gumede-Moyo S, Filteau S, Munthali T, Todd J, Musonda P. Implementation effectiveness of revised (post-2010) World Health Organization guidelines on prevention of mother-to-child transmission of HIV using routinely collected data in sub-Saharan Africa: A systematic literature review. Medicine (Baltimore). 2017;96(40):e8055.
7. Russell A, Hellawell G. The future of electronic health records. Br J Hosp Med (Lond). 2013;74(11):604-5.
8. Namulanda G, Qualters J, Vaidyanathan A, Roberts E, Richardson M, Fraser A, et al. Electronic health record case studies to advance environmental public health tracking. J Biomed Inform. 2018;79:98-104.
9. Cifuentes M, Davis M, Fernald D, Gunn R, Dickinson P, Cohen DJ. Electronic Health Record Challenges, Workarounds, and Solutions Observed in Practices Integrating Behavioral Health and Primary Care. J Am Board Fam Med. 2015;28 Suppl 1:S63-72.
10. Fraser HS, Biondich P, Moodley D, Choi S, Mamlin BW, Szolovits P. Implementing electronic medical record systems in developing countries. Inform Prim Care. 2005;13(2):83-95.
11. Allen C, Jazayeri D, Miranda J, Biondich PG, Mamlin BW, Wolfe BA, et al. Experience in implementing the OpenMRS medical record system to support HIV treatment in Rwanda. Stud Health Technol Inform. 2007;129(Pt 1):382-6.
12. Siika AM, Rotich JK, Simiyu CJ, Kigotho EM, Smith FE, Sidle JE, et al. An electronic medical record system for ambulatory care of HIV-infected patients in Kenya. Int J Med Inform. 2005;74(5):345-55.
13. Oluoch T, Santas X, Kwaro D, Were M, Biondich P, Bailey C, et al. The effect of electronic medical record-based clinical decision support on HIV care in resource-constrained settings: a systematic review. Int J Med Inform. 2012;81(10):e83-92.
14. Singh J, Filteau S, Todd J, Gumede-Moyo S. Progress in the performance of HIV early infant diagnosis services in Zambia using routinely collected data from 2006 to 2016. BMC public health. 2018;18(1):1297.
15. Gumede-Moyo S, Todd J, Schaap A, Mee P, Filteau S. Effect of prevention of mother-to-child transmission strategies on antiretroviral therapy coverage in pregnant women in Zambia: analysis using routinely collected data (2010–15). Lancet Global Health. 2019;7(Supplement 1):S25.
16. Muyunda G. Zambia leads the way in SmartCare electronic health records system, a benefit to both providers and patients 2011 [Available from:

For peer review only - http://bmjopen.bmj.com/site/about/guidelines.xhtml

https://www.jhpiego.org/success-story/zambia-leads-the-way-in-smartcare-electronic-health-records-system-a-benefit-to-both-providers-and-patients/.
17. Lincoln NKDYS. Handbook of Qualitative Research 2nd ed. London: Sage Publications; 2000.
18. Vaismoradi M, Turunen H, Bondas T. Content analysis and thematic analysis: Implications for conducting a qualitative descriptive study. Nurs Health Sci. 2013;15(3):398-405.
19. Graneheim U.H. LB. Qualitative Content Analysis in Nursing Research: Concepts, Procedures and Measures to Achieve Trustworthiness. Nurse Education Today 2004;24:105-12.
20. Mamlin BW, Biondich PG. AMPATH Medical Record System (AMRS): collaborating toward an EMR for developing countries. AMIA Annu Symp Proc. 2005:490-4.
21. Della Mea V. Internet electronic mail: a tool for low-cost telemedicine. J Telemed Telecare. 1999;5(2):84-9.
22. Abiy R, Gashu K, Asemaw T, Mitiku M, Fekadie B, Abebaw Z, et al. A Comparison of Electronic Medical Record Data to Paper Records in Antiretroviral Therapy Clinic in Ethiopia: What is affecting the Quality of the Data? Online journal of public health informatics. 2018;10(2):e212.
23. Fritz F, Tilahun B, Dugas M. Success criteria for electronic medical record implementations in low-resource settings: a systematic review. J Am Med Inform Assoc. 2015;22(2):479-88.
24. Williams F, Boren SA. The role of the electronic medical record (EMR) in care delivery development in developing countries: a systematic review. Inform Prim Care. 2008;16(2):139-45.
25. Ivers NM, Sales A, Colquhoun H, Michie S, Foy R, Francis JJ, et al. No more 'business as usual' with audit and feedback interventions: towards an agenda for a reinvigorated intervention. Implementation science : IS. 2014;9:14.
26. Hemler JR, Hall JD, Cholan RA, Crabtree BF, Damschroder LJ, Solberg LI, et al. Practice Facilitator Strategies for Addressing Electronic Health Record Data Challenges for Quality Improvement: EvidenceNOW. J Am Board Fam Med. 2018;31(3):398-409.
27. Douglas GP, Gadabu OJ, Joukes S, Mumba S, McKay MV, Ben-Smith A, et al. Using touchscreen electronic medical record systems to support and monitor national scale-up of antiretroviral therapy in Malawi. PLoS medicine. 2010;7(8).

For peer review only

For peer review only - http://bmjopen.bmj.com/site/about/guidelines.xhtml

Table 1: Breakdown of In-depth Interviews and Focus Group Discussions Participants

**Type of respondents**	**Number of respondents**	**Method**
Leadership	3	In-depth interviews
Database Management	6	
Data Entry Clerks	5	
MCH- Nurse-in charge	3	
**Total**	**17**	
Pregnant Mothers	(>20 years) 2	Focus Group Discussions
	(20-25years) 3	
	(26-30 years) 4	
	(31-35 years) 2	
	(36-40 years) 1	
	**Total 12**	
Lactating Mothers	(>20 years) 1	
	(20-25years) 2	
	(26-30 years) 2	
	(31-35 years) 3	
	(36-40 years) 2	
	**Total 10**	

For peer review only

Figure 1: SmartCare Model Structure

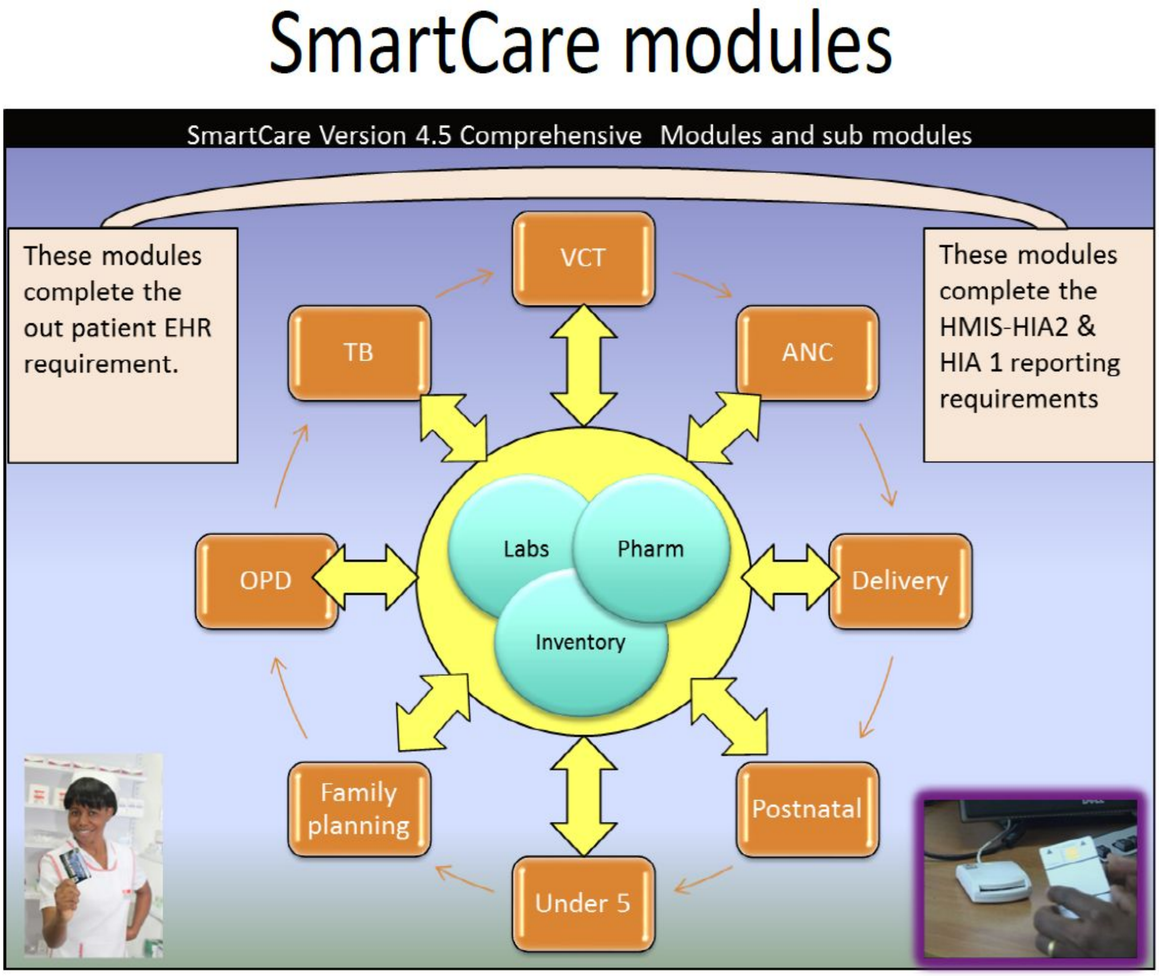

w only

Figure 2 - Breakdown of In-depth interviews key informants

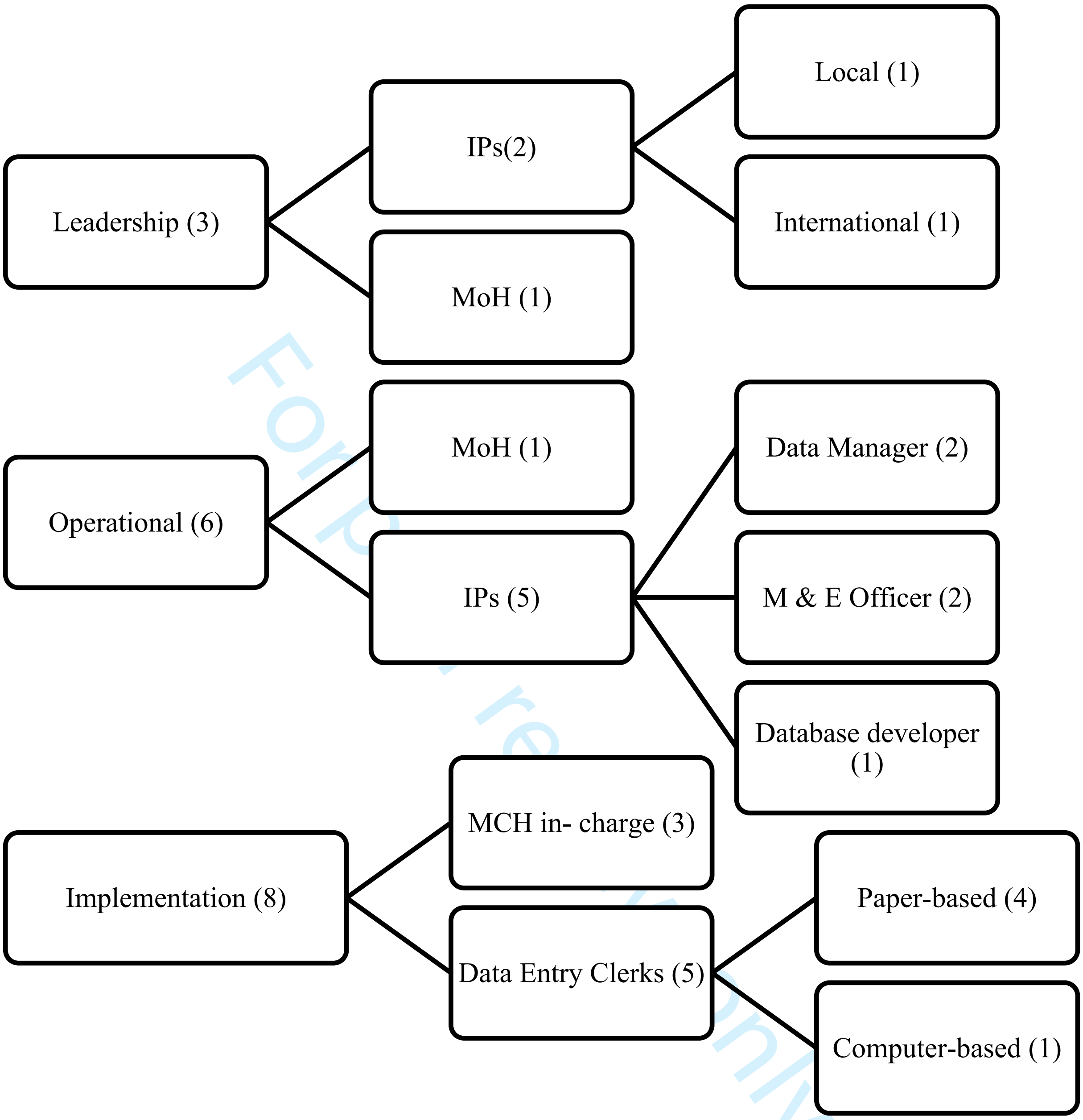

Figures in brackets are for the number of people interviewed for that designated level.

Figure 3: SmartCare Data flow

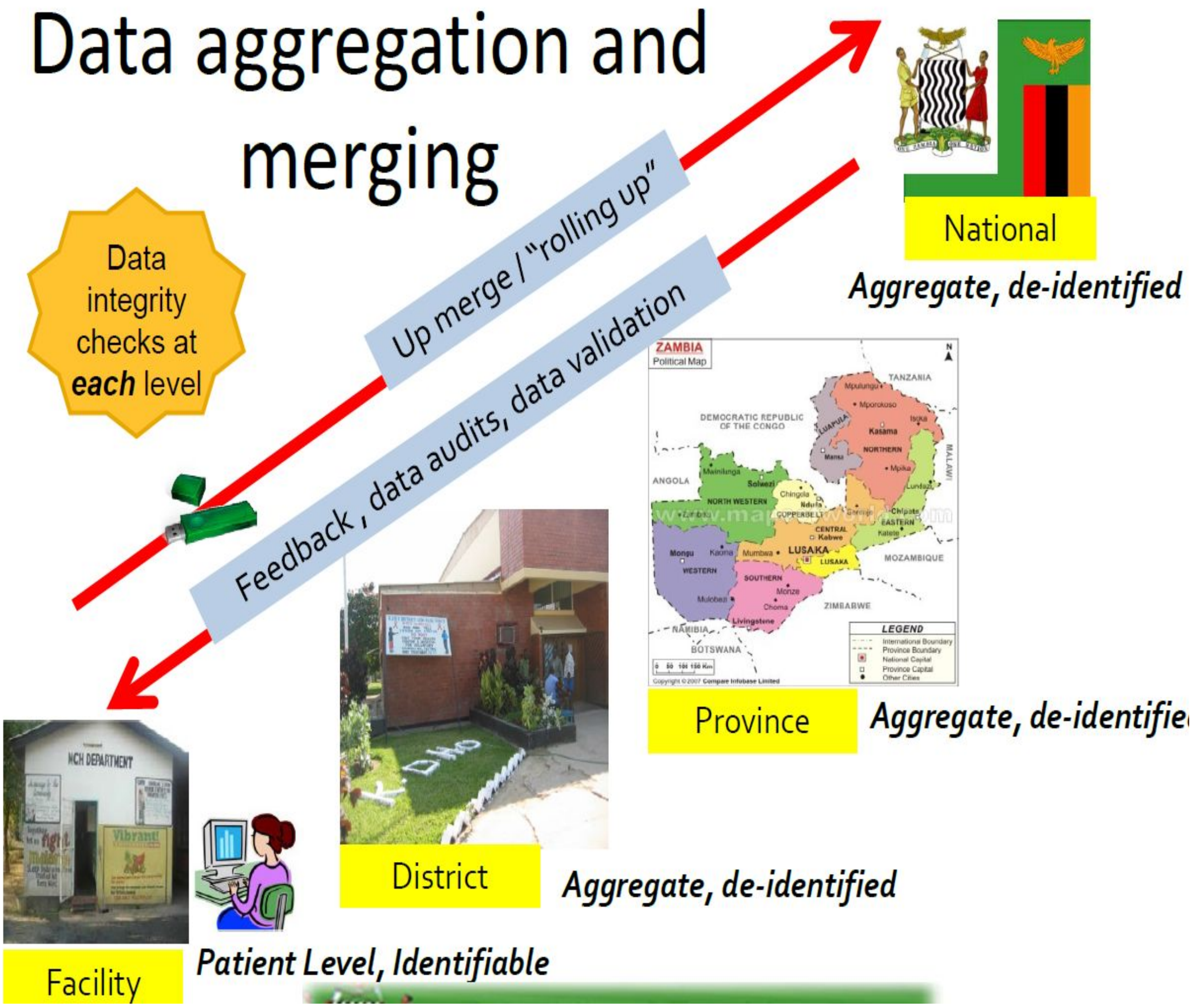

COREQ (COnsolidated criteria for REporting Qualitative research) Checklist

A checklist of items that should be included in reports of qualitative research. You must report the page number in your manuscript where you consider each of the items listed in this checklist. If you have not included this information, either revise your manuscript accordingly before submitting or note N/A.

Topic	Item No.	Guide Questions/Description	Reported on Page No.
**Domain 1: Research team and reflexivity**			
Personal characteristics			
Interviewer/facilitator	1	Which author/s conducted the interview or focus group?	7
Credentials	2	What were the researcher's credentials? E.g. PhD, MD	1
Occupation	3	What was their occupation at the time of the study?	1
Gender	4	Was the researcher male or female?	N/A
Experience and training	5	What experience or training did the researcher have?	1
Relationship with participants			
Relationship established	6	Was a relationship established prior to study commencement?	4
Participant knowledge of the interviewer	7	What did the participants know about the researcher? e.g. personal goals, reasons for doing the research	6
Interviewer characteristics	8	What characteristics were reported about the inter viewer/facilitator? e.g. Bias, assumptions, reasons and interests in the research topic	6-7
**Domain 2: Study design**			
Theoretical framework			
Methodological orientation and Theory	9	What methodological orientation was stated to underpin the study? e.g. grounded theory, discourse analysis, ethnography, phenomenology, content analysis	5
Participant selection			
Sampling	10	How were participants selected? e.g. purposive, convenience, consecutive, snowball	6-7
Method of approach	11	How were participants approached? e.g. face-to-face, telephone, mail, email	7
Sample size	12	How many participants were in the study?	
Non-participation	13	How many people refused to participate or dropped out? Reasons?	6
Setting			
Setting of data collection	14	Where was the data collected? e.g. home, clinic, workplace	7
Presence of non-participants	15	Was anyone else present besides the participants and researchers?	7-8
Description of sample	16	What are the important characteristics of the sample? e.g. demographic data, date	6 & 7
Data collection			
Interview guide	17	Were questions, prompts, guides provided by the authors? Was it pilot tested?	7
Repeat interviews	18	Were repeat inter views carried out? If yes, how many?	17
Audio/visual recording	19	Did the research use audio or visual recording to collect the data?	7-8
Field notes	20	Were field notes made during and/or after the inter view or focus group?	8
Duration	21	What was the duration of the inter views or focus group?	7
Data saturation	22	Was data saturation discussed?	N/A
Transcripts returned	23	Were transcripts returned to participants for comment and/or	N/A

For peer review only

Topic	Item No.	Guide Questions/Description	Reported on Page No.
		correction?	
**Domain 3: analysis and findings**			
Data analysis			
Number of data coders	24	How many data coders coded the data?	8
Description of the coding tree	25	Did authors provide a description of the coding tree?	9
Derivation of themes	26	Were themes identified in advance or derived from the data?	8
Software	27	What software, if applicable, was used to manage the data?	8
Participant checking	28	Did participants provide feedback on the findings?	N/A
Reporting			
Quotations presented	29	Were participant quotations presented to illustrate the themes/findings? Was each quotation identified? e.g. participant number	8
Data and findings consistent	30	Was there consistency between the data presented and the findings?	N/A
Clarity of major themes	31	Were major themes clearly presented in the findings?	9-15
Clarity of minor themes	32	Is there a description of diverse cases or discussion of minor themes?	15-17

Developed from: Tong A, Sainsbury P, Craig J. Consolidated criteria for reporting qualitative research (COREQ): a 32-item checklist for interviews and focus groups. *International Journal for Quality in Health Care*. 2007. Volume 19, Number 6: pp. 349 – 357

Once you have completed this checklist, please save a copy and upload it as part of your submission. DO NOT include this checklist as part of the main manuscript document. It must be uploaded as a separate file.

For peer review only

BMJ Open

A Qualitative Inquiry into Implementing an Electronic Health Record System (SmartCare) for Prevention of Mother-to-Child Transmission data in Zambia

Journal:	*BMJ Open*
Manuscript ID	bmjopen-2019-030428.R1
Article Type:	Research
Date Submitted by the Author:	28-May-2019
Complete List of Authors:	Gumede-Moyo, Sehlulekile; London School of Hygiene and Tropical Medicine, Epidemiology and Population Health<br>Todd, Jim; London School of Hygiene and Tropical Medicine Faculty of Epidemiology and Population Health<br>Bond, Virginia; London School of Hygiene and Tropical Medicine Faculty of Epidemiology and Population Health<br>Mee, Paul; London School of Hygiene and Tropical Medicine Faculty of Epidemiology and Population Health<br>Filteau, Suzanne; London School of Hygiene and Tropical Medicine Faculty of Epidemiology and Population Health
**Primary Subject Heading**:	Health informatics
Secondary Subject Heading:	Health services research, Qualitative research
Keywords:	PMTCT, routinely collected data, electronic health records

SCHOLARONE™
Manuscripts

A Qualitative Inquiry into Implementing an Electronic Health Record System (SmartCare) for Prevention of Mother-to-Child Transmission data in Zambia

Sehlulekile Gumede-Moyo (BSc, MPH, MBA, PhD,)[1,2§]; Jim Todd (MA, MSc)[1] ; Virginia Bond (MSc, PhD)[1,3] Paul Mee (BSc, MSc, PhD)[1] and Suzanne Filteau (BSc, MSc, PhD)[1]

1. Department of Population Health, Faculty of Epidemiology and Population Health, London School of Hygiene and Tropical Medicine, London, UK

2. School of Public Health, University of Zambia, Lusaka, Zambia

3. ZAMBART, University of Zambia, School of Medicine

[§]Corresponding author: Sehlulekile Gumede-Moyo. Department of Population Health, Faculty of Epidemiology and Population Health, London School of Hygiene and Tropical Medicine, Keppel Street, London WC1E 7HT, UK. (sehlulekile.gumede@lshtm.ac.uk)

Email addresses of authors:

SGM: sehlulekile.gumede@ lshtm.ac.uk

JT: jim.todd@lshtm.ac.uk

VB: virginia.bond@lshtm.ac.uk

PM: paul.mee@ lshtm.ac.uk

SF: suzanne.filteau@lshtm.ac.uk

For peer review only

Key words: PMTCT, routinely collected data, electronic health records, implementation.

Abstract

Introduction: Electronic Health Records (EHR) could present an opportunity to improve accuracy, timeliness and scope of surveillance data. This study aimed to investigate the challenges in implementing a Zambian EHR system labelled 'SmartCare' from diverse stakeholder perspectives in order to improve PMTCT data collection so that SmartCare can be used for clinic performance strengthening and program monitoring.

Method: Data were collected using in-depth interviews, observations and focus groups discussions (FGD) between September and November 2016. Seventeen in-depth interviews were held with a range of key informants from the Ministry of Health and local and international organisations implementing SmartCare. Four data entry observations and three FGDs with 22 pregnant and lactating women seeking PMTCT services were conducted. Data were analysed using a thematic content approach.

Results: The SmartCare system has evolved from various patient tracking systems into a multifunctional system. There is a burden of information required so that sometimes not all is collected and entered into the database, resulting in poor data quality. Funding challenges impede data collection due to manpower constraints and shortages of supplies. Challenges associated with data collection depend on whether a paper- or computer-based system is utilised. There is no uniformity in the data quality verification and submission strategies employed by various implementing partners. There is little feedback from the EHR system at health facility level, which has led to disengagement as stakeholders do not see the importance of the system.

Conclusion: SmartCare has structural challenges which can be traced from its development. Funding gaps have resulted in staffing and data collection disparities within implementing partners. The lack of feedback from the system has also led to complacency at the operational level which has resulted in poor data quality in later years.

(297 words)

For peer review only - http://bmjopen.bmj.com/site/about/guidelines.xhtml

Strengths

- We obtained a rich and nuanced appreciation of the implementation of the SmartCare as we sampled from a range of IPs, health facilities, stakeholders and implementation systems.
- The depth of the inquiry enabled us to consider a range of explanatory factors.

Weaknesses

- Data collection was restricted to health facilities in and around Lusaka, the capital city of Zambia, and therefore the health facilities selected could be receiving financial and technical support of an order that is unlikely to be offered in remote facilities.

For peer review only

Introduction

Routinely collected clinic data can provide much needed information on the prevalence of HIV among pregnant women and the uptake of services for prevention of mother-to-child transmission (PMTCT) of HIV(1). The use of routinely collected data can be timely and cost-efficient for decision making as data are already available for analysis (2). Collection of high-quality routine data on these services and outcomes for HIV-positive mothers and HIV-exposed infants is essential for monitoring and evaluation of PMTCT programs, for clinical management of patients, and for managing stocks of HIV test kits and drugs (1). For both clinic staff and health system managers, having access to reliable data that reflects the processes of care and clinical outcomes is the first step to ensuring effective delivery of an intervention within a health care system(3, 4). However in Zambia there is underutilization of routinely collected data in the HIV programs (5).

Data collection systems for PMTCT programs in Africa often lack immediacy as many are paper-based with records completed at each facility, and with individual level data that are inaccessible to central planners(6). Electronic health records (EHRs) could present an opportunity to supplement current sources of routinely collected surveillance data (7).EHRs are real-time, patient-centred records that make information available instantly and securely to authorized users (8). While an EHR does contain the medical and treatment histories of patients, an EHR system is built to go beyond standard clinical data collected in a provider's office and can include a broader view of a patient's care (9).

Various EHR systems are implemented globally (10-12). A systematic of review of literature about EHR systems in resource-constrained settings recommended that it is urgent to evaluate barriers to implementation (13). Our own research on extracting surveillance data from a Zambian EHR system, SmartCare, has highlighted some deficiencies including large amounts of missing data, especially in more recent years, and variable performance across the country (14, 15). This study aimed to investigate the challenges in implementing SmartCare from diverse stakeholder perspectives in order to improve PMTCT data collection so that it can be used for clinic performance strengthening and program monitoring.

For peer review only - http://bmjopen.bmj.com/site/about/guidelines.xhtml

Methods

Study design

This qualitative study included in-depth interviews, observations and focus groups discussions (FGDs) conducted between September and November 2016.

Study Setting

SmartCare is a Zambian Ministry of Health (MoH)-led project funded by the United States Centre for Disease Control and Prevention (CDC). SmartCare was developed to improve continuity of care and provide timely data on maternal and child health, HIV/AIDS, tuberculosis and malaria interventions for public health purposes, trend reporting and analysis for health officials and clinicians (16). The Implementing partners (IPs) are responsible for data entry mainly from paper-based forms which have information collected by clinicians which is then entered into a computer.

The SmartCare database is a derivative of the PTS (patient tracking system) which was developed in 2004, based on a health facility-centred EHR system. In 2010, it became a national medical health program and was then rolled out throughout the country. It is implemented by government, and both international and local organisations primarily for patient management. Most of the international organisations were involved in the database development, and the local organisations became involved when implementation was rolled out throughout the country (after 2009).

SmartCare is organised into comprehensive modules and sub-modules ***(Figure 1).*** This was mainly influenced by its funders depending on the information they wanted at that particular time. The main module groups are: clinical group for which the modules of interest in this study are Antenatal care(ANC), Delivery, Postnatal, Antiretroviral therapy (ART), Paediatric ART, PMTCT follow up, and Under 5; logistic group which has information on drug dispensing and orders; monitoring and evaluation groups which includes health management information system reports, graphing, data analysis, data merge from facilities for MOH; and the continuity of care group which has data from across facilities and within facilities. Through the data-merge the SmartCare information can be used to obtain data for the monthly reports to the MOH.

For peer review only - http://bmjopen.bmj.com/site/about/guidelines.xhtml

Data is captured under a number of 'modules' across a range of health issues. Records are updated at every point of clinical service. Patients are issued with Smartcards at their initial consultation which contains all their clinical information and treatment details and can be accessed from any SmartCare facility.

The SmartCare data is collected either directly onto a computer or using a paper-based method. When using the computer-based data collection and entry method, clinicians have to enter data directly in the computer; this method is still being piloted in a few facilities mainly in the Southern and Lusaka provinces. For the more common paper-based data method, client information is documented in the SmartCare care forms. The papers are then taken to the data entry room where the information is entered into the computer. Some facilities which are deep rural (where there is no electricity at all) use paper only and the forms are then transferred to a location where there are computers and entered into the database.

Data on a range of HIV and pregnancy-related outcomes are collected during the patient consultation with the clinician, who records the information on paper forms or directly on the computer in the few facilities which are computer-based. The paper forms are then entered into the SmartCare database by data entry clerks who have been trained in using SmartCare. Data is collected from each facility on a monthly basis and submitted to a District Health Information Officer, who aggregates and sends the data to the province level Senior Health Information Officers. From here the data are wired to a national server at MoH headquarters ***(Figure 2).***

For the purposes of this study, routine data are defined as data that are routinely generated through ANC and PMTCT service delivery, and routinely recorded in standard SmartCare data tools.

Sampling

Data were generated from 17 in-depth semi-structured face-to-face interviews, four data entry observations and three FGDs with 22 pregnant and lactating women seeking PMTCT services from three health facilities (Table 1).

Key informant interviews

The IPs were purposively sampled in consultation with the MoH to identify diverse organizations. Six health facilities were selected based on local considerations which

For peer review only - http://bmjopen.bmj.com/site/about/guidelines.xhtml

included: both well-resourced international organizations and less well-resourced local and government-owned organizations; those best performing versus struggling; those which are paper- or computer-based; and facilities which had already transitioned to ‘test and treat’ for general population HIV care. Most of the facilities sampled were in Lusaka City, and only one was peri-urban. The choice of Lusaka was based on the following considerations: 1) logistics, 2) facilities in Lusaka are busy and have the highest case loads, 3) our previous work has shown that facilities in Lusaka show a wide range of successes and failures, and 4) overall Lusaka performs poorly compared to the rest of the country and therefore it was important to investigate the working of SmartCare in Lusaka city.

Within each of the selected IPs, a diverse range of interviewees were purposively recruited in order to give the broadest range of perspectives (17, 18). The approaches to recruitment of participants were flexible, being negotiated with key contacts and recommendations from managers who were also considered for in-depth interviews. Interviews with key informants were conducted by the first author in English. They lasted 30 to 60 minutes and consisted of a series of open-ended questions with follow-up question probes. The interview guides were specific to the hierarchical levels of operation, namely: leadership level (persons involved in the strategic direction of SmartCare); operational level (Data Managers and Monitoring and Evaluation personnel); and implementation level (Data entry clerks and MCH in-charge clinicians). The interview guides were designed to capture information on SmartCare development, implementation procedures, data quality management and linkage between SmartCare and PMTCT services.

Focus group discussions

The FGDs were conducted at maternal and child health (MCH) departments with participants from three different facilities, two from Lusaka and one from the peri-urban area. The participants were recruited with the assistance of PMTCT coordinators who were instructed to balance the number of pregnant and lactating women on ART. The FGD sessions were conducted by the first author and a co-moderator who was fluent in the local languages (Bemba and Nyanja). These discussions were guided by an interview guide containing open-ended questions and lasted for 60 to 90 minutes. The interview guide was designed to capture the general knowledge and experience of the PMTCT program and SmartCare operations.

For peer review only

For peer review only - http://bmjopen.bmj.com/site/about/guidelines.xhtml

Facility observations

The observations were conducted to familiarise the researcher with the realities of the SmartCare data collection and entry environments. These were planned to be conducted in 6 health facilities through interaction with study participants and relevant actors as well as personal reactions to the related events without the use of a specific tool. However in 2 health facilities data entry was not taking place on the scheduled visit days due to power outage, hence only in-depth interviews were conducted with implementing level staff.

2.5 Data Management and Analysis

The in-depth interviews and FGDs were tape-recorded and subsequently transcribed verbatim. FGDs were then translated into English. Data were collected aiming to get sufficient data from relevant actors/perspectives to ensure it was qualitatively representative(19). Detailed field notes were taken on the sites following the observations for analysis.

A thematic content approach was conducted; this was done by reading the transcribed material systematically, line by line, in order to identify the meaning units. Meaning units were defined as strings of the text expressing a single coherent thought, up to the point at which the coherent thought changed. Thereafter, the meaning units were marked by a code, describing what the text unit was about. Coding was also done on issues that were related to the reasons for missing PMTCT data; text unrelated to these issues was not included in the present analysis.

The codes were defined and organised so that those referring to the same subject were grouped into categories. The interview guide was used as a starting point for grouping the information, but during the analysis new categories were developed. When this happened, we re-coded. The data were managed manually in Excel sheets. The underlying meanings of the categories were formulated into a theme. The original data were re-assessed by an assistant (MSc Student) after analysis in order to detect any concepts or information that would have been missed. The first author discussed the results with SF, JT and the MSc student.

3. Research ethics

Written informed consent was obtained from all key informants including their consent to record the interviews and publish anonymous quotations. Verbal consent was sought for FGD participants. Participation in the study was entirely voluntary and participant anonymity was maintained throughout the processes of interview transcription, data analysis and presentation

For peer review only - http://bmjopen.bmj.com/site/about/guidelines.xhtml

by using pseudonyms. Permission to collect data from the health facilities was granted by the MoH district offices. The study was approved by the Zambia Biomedical Ethical Board (Ref 101-04-16) and the LSHTM Research Ethics Committee (Ref 12086).

4. Results

The comments from key informants, observations and the focus group discussions enabled us to characterise four fundamental issues that are key in the implementation of SmartCare: database development and ownership, funding and staffing, the data collection process, and health facility set up.

4.1 Database Development and Ownership

The development of SmartCare was mainly CDC-led, with the government of Zambia through the MoH supporting the process. Multiple stakeholders using various patient tracking software were brought together to form SmartCare. Reflecting on the history of SmartCare, two IP informants identified the leading role of CDC and the subsequent collaboration with a broader group of partners.

'The initial development was by the American-funded institutions and a few people from the MoH because they had gone into partnership. They were developers at MoH and also at CDC, the rest of the institutions like us were only required to use the software.'- Implementing partner leader.

'There were other systems that were used by other partners throughout the country e.g., there was a system called the CareWare which was similar to SmartCare but it was just a patient summary. Then there was another system which was used in the private sector which l can't remember the name. Lessons were drawn from all these systems and brought into one and this is how SmartCare was born.'- Implementing partner leader.

There was a consensus among the leaders of the SmartCare implementation that the database has also evolved from a system to track patients to its current form as a mixture of a patient tracking tool, a clinical care tool, a reporting tool, and a surveillance tool.

'...it was just a mixture of ideas which were clinically required but each person had different needs for information, some needed it for research purposes, l needed it for patient follow up, another person needed it for forecasting and quantification'- Implementing partner leader.

For peer review only - http://bmjopen.bmj.com/site/about/guidelines.xhtml

The different stakeholders had different data needs which took the form of added modules. The changes have however, made it difficult to classify and use the database because it now tries to address multiple issues simultaneously.

*'I wanted my things as well and partly l wanted things that PEPFAR (*The United States President's Emergency Plan for AIDS Relief) *wanted because our money comes from them. If I can't report on PEPFAR indicators, l don't get more money' –Implementing partner leader.*

The resulting SmartCare forms are 6-7 pages for a normal interaction per visit because of requirements from its various stakeholders. In addition, a lot of data has been entered into the system making it bulky. This has caused the system to become very slow, and in the mornings it takes a very long time to reboot as narrated by operational and implementing level participants.

'Because the system is slow they have to wait instead of entering 100 files per day they are only able to enter 60 or so.'- Database Manager.

Data entry is often interrupted as the system also hangs up every now then because it is so bulky. Hence a lot of backlogs are experienced which results in a lot of data not being entered.

'Last year it had serious faults and we were not using it for a while.' – Data Entry Clerk.

Although there is a SmartCare technical working group, chaired by the MoH where operations are discussed, it is not clear who is guiding the process, and how the database should be modified to make it work better.

4.2 Funding and staffing

The database operations are managed by the monitoring and evaluation (M&E) departments of the IPs. The structures of the M&E departments differ across the IPs, with international organisations having much bigger and better resourced departments. The local IPs have much leaner structure, rely mostly on volunteers, and run into problems due to financial constraints. This was elaborated by the leadership and implementing level participants.

'Mostly the people who enter data in the health facilities where we operate are employed by us. Apart from that we also provide support through volunteers; in most cases we also provide peers and counsellors. –Data Base Manager.

For peer review only

The manpower constraint in the local partners has resulted in the loss of data during long grant negotiations. Data is lost as data entry staff are laid off between funding cycles since their salaries are dependent on grants. In addition, these partners have designated personnel who enter general ART data whilst PMTCT data is mainly entered when the data entry personnel have specific interest or have trained temporary volunteer staff.

'The positions of data entry clerks in the facilities are actually paid for by the donor. It's not sustainable because the moment the donor says we are pulling out, there will not be any data entry personnel in these facilities.'- Implementing partner leader.

The funding of operations also affects the supplies required for the day-to-day operations as narrated by both operations and implementing personnel.

'We normally run out of forms, especially when funding is out. When we don't have funding the facilities also have challenges in printing the forms because toner is expensive.'- Data Entry Clerk.

'….when the computers are down because of the virus, data entry doesn't happen.'- M &E Officer.

In some small facilities where the MoH is solely responsible for data collection and entry, there are no data entry clerks and hence clinicians enter the data on the computer after hours. This is usually done only by staff that are passionate and so give it time according to a MoH leader.

'In the government facilities there is no designated person to manage ART data for SmartCare; its health workers who just do that on their own. - 'Implementing partner leader.'

4.3 Data Collection Process

4.3.1 Data entry

With both the computer and the paper-based methods the greatest challenge is incomplete forms which translate to missing data in the database. In some facilities which are computer-based, the data entry clerks noticed that there are also some clinicians who still prefer entering data on paper and handing over to the data entry clerks to enter on the computer.

For peer review only - http://bmjopen.bmj.com/site/about/guidelines.xhtml

'In as much as data is supposed to be entered at each service point, some people prefer entering the data on paper and they hand over the data to me.'- Data Entry Clerk

According to implementing level staff, with the paper-based method, once the client leaves the facility it is a challenge to follow up on the missing data on the forms.

'There are usually many gaps in the forms; the forms are not fully completed more than half of the time.'- Data Entry Clerk.

'When the forms are not complete l return them to whoever was supposed to be entering the data at that service point but the challenge is that they will tell you the person has gone and that can't follow them up and even so in the next visit they will not even bother correcting the information'- Data Entry Clerk.

One of the MoH participants explained that the incomplete data forms could be a result of the competing demands to physically attend to clients and fill out the forms in the high volume facilities.

'.... It takes 20-30 minutes just to enter the data for one interaction. In clinics where there are many patients, how many patients are you going to see in a day?' – Implementing partner leader.

The implementation challenges of the SmartCare database depend on the data collection and entry method used. Power outages were the greatest challenge: under the computer-based method, client information is not collected, as some clinicians will be reluctant to use the alternative method, whereas with the paper-based method there will be a backlog of work.

Some well-funded partners have installed solar power systems in the health facilities, and this enables them to enter data without interruptions. Despite having good power supply, there is a challenge in these facilities of some clinicians who are not computer literate. Furthermore, because of other competing health facility priorities, the power is rarely used for data entry.

'......would you want to power theatre activities or you want to power computers for data entry? Theatre is life saving and data can be entered anytime.' – Implementing partner leader.

For peer review only - http://bmjopen.bmj.com/site/about/guidelines.xhtml

4.3.2 Data quality verification

Verification of data quality is specific to each implementing partner. The verification process involves comparing the patient information on the paper forms and registers against the information in the database. The international partners also have verification procedures imbedded in the Monitoring and Evaluation strategies as narrated by one of their participants.

'We are doing double data entry for 10% of our files.' – Data Manager.

On the other hand, verification is rarely done by the local organisation due to manpower constraints as indicated by an operational level participant.

'When l get time, l check what is in the system versus what is in the registers. However it's rare that l get that time because l always have files to enter into the system. In that case l wait for the Quality Control people from Head Office to come and do random verifications for me.'- Data entry clerk

All the partners reported that there are some instances where they can't find patient files, making it difficult for them to verify the data that is in the computer with the raw data.

'There are certain instances where we can't find patient files'. – M&E Officer.

The patient files will tell me that a facility has 200 clients whilst SmartCare has 300 clients; this is because some of these files are kept somewhere, could be files of staff members or people with prominent positions in that particular area'. – Database Manager

The SmartCare Database system has reports that are built in the application. However all the partners alluded that these reports are very inconsistent and incorrect.

'In some cases it will show a large number of people who would have been initiated in a day more than what we would have actually done.'– MCH Nurse in Charge.

4.3.3. Submission of data to the main database

There is also no uniformity in submission of the data to the main database, although according to the MOH the data is supposed to flow from the facility to the district, province and then to the main database at head office (Figure 2).

'They sometimes come to the facilities and collect the data on their own since they are the ones who own these facilities as well' – M & E officer.

For peer review only - http://bmjopen.bmj.com/site/about/guidelines.xhtml

Some partners wait for MoH to request data whilst some submit their data to CDC.

'We do not follow that procedure as per say because we collect data directly from the sites and aggregate it from here and submit to CDC. If MOH requests it, we submit to them but we are not that consistent'- Database Manager

4.3.4Feedback from Database

There is no standardised procedure for reporting data that is collected by IPs. The partners normally use the information internally as well as sharing with the donors supporting them.

'We also have special reports that we do in a monthly basis such as the PEPFAR report' – M & E Coordinator.

In contrast, the nurses-in-charge in some of the facilities reported that they have never received feedback from the system.

'We have not received anything; they don't give us any reports. We would, however, want to receive reports so that we would know how we are faring.' – MCH Nurse in Charge.

As viewed by one of the leaders who participated in the study, the lack of feedback from the system has led to disengagement and some stakeholders not seeing the importance of documenting client information in the SmartCare Database.

'*A lot of the staff collecting this data do not understand how and why they are collecting this data and they are frustrated by it as it also takes lots of their time entering this data' – Implementing partner leader.*

4.4 Health Facility setup

The setup in some health facilities is in such a way that the PMTCT services are offered in the ANC department whereas ART services are offered in places designated solely for ART. The nurses in charge in such health facilities narrated that the facility setup could be a great challenge in ensuring that the PMTCT data is entered in an efficient manner.

'Probably if it was housed within the ART department, you could have the same person entering the PMTCT data into the SmartCare' – MCH nurse in charge.

In addition, the MCH nurses-in-charge indicated that client files sometimes go missing between the MCH and the data room.

For peer review only - http://bmjopen.bmj.com/site/about/guidelines.xhtml

'I am sure when they are sending the files; some are lost during transfer in different departments.' – MCH nurse-in-charge.

Views of pregnant and lactating women

Overall, women seeking PMTCT services did not have concerns on the implementation of SmartCare, as according to them the healthcare workers will be doing their job.

'The person who is supposed to enter the data does so because he was trained anyway'- PMTCT FGD Mothers.

'We just carry our cards and leave them to do their job'- PMTCT FGD Mothers.

'We are here for our lives and that of our babies, we have to comply and be patient'- PMTCT FGD Mothers

5. Discussion

The study has provided evidence on the SmartCare implementation challenges. It has also provided insights as to how to improve implementation, and data quality. The main points were to collect less, but better data, to engage the clinic staff by providing regular feedback, and to improve the software. Fortunately, PMTCT clients appear already satisfied with the system.

The design of EMR systems does not always get attention due to pressures related to their functionality requirements(10). SmartCare was exposed to pressures as its data model evolved from a patient tracking system into a multi-functional system due to demands of various stakeholders. In an effort to shape the SmartCare database into a manageable tool that can be used for public health purposes, CDC and MOH could streamline the process for including and excluding information that is collected. There is a need to cut down on the number of fields collected so that less data of higher quality data is collected. This could improve the system by: reducing the amount of data so that it takes less time to collect and people might more readily do it; and having a less bulky database which will be less likely to be affected by computer crashes and faster to use.

For peer review only - http://bmjopen.bmj.com/site/about/guidelines.xhtml

For peer review only

SmartCare could be developed into a networked EMR, which has been reported to be successful in other developing countries like Rwanda, and Kenya (23). In a networked EMR data can be accessible and be shared in multiple sites; multiple users can enter data simultaneously; data can be backed up automatically at more than one site; information can be communicated between multiple locations; data are accessible and shared at multiple sites and the system can be debugged and upgraded over the internet without visiting remote sites (10, 24). This would allow greater use of the data by health workers and Ministry planners. However, it is important that there is uniformity in the implementation of SmartCare throughout the country so that everyone will be on the same level. This will allow for comparisons across geographic regions and longitudinal analyses.

In Ethiopia the quality of data were reported to be affected by dual documentation where both paper based and electronic systems were used (20). According to Odekunle et al limited computer skills is one of the factors affecting electronic health record adoption in sub-Saharan Africa. The authors pointed out that in some countries, many physicians and other key end users are not eager to adopt an EHR, resulting in low EHR adoption in the region (21).There was a notable lack of appreciation of the system, and a need to train and support end users of the system such as the clinicians who are directly involved in the data collection process. For both the clinic staff and health system managers, having access to reliable data that reflects the processes of care and clinical outcomes is the first step to ensuring effective delivery of an intervention within a health care system(4). Hence, there should be better buy-in of the staff at the facilities in order to make sure that they fill in the forms regularly and accurately.

In our findings workload affected the documentation, as evidenced by poor data quality in the quantitative analysis of SmartCare PMTCT data (14, 15). In low–resourced countries there is a shortage of a qualified work-force and most health care institutions do not have dedicated IT staff for their EMR systems (22). The results of our study are consistent with a systematic review of literature on the role of EHR systems in developing countries which pointed to the lack of financial and human resources as major challenges in implementing EMR systems (23). Therefore, there is a great need for strong commitment from both the government and the donors to invest in improving the implementation of the system.

For peer review only - http://bmjopen.bmj.com/site/about/guidelines.xhtml

The data from the database should be presented to facility staff as an advocacy tool which is needed in their facilities, so that they would appreciate the need for it (24, 25). Similarly, in their description of the rationale and experience in scaling up EMR in Malawi, Douglas and colleagues concluded that health workers will not adopt a system if they do not find sufficient value in it (26).It is also possible that health workers perceive SmartCare as complex, and of little value to their work; therefore any revision to the system would require a clear strategy for ensuring that the operations are well conceptualized (27).

The knowledge required for the use of the database should easily be transferred through training, ensuring that it is customized to the IPs' requirements and the establishment of helpdesk call centres to readily assist with operational challenges. There is also a need to strengthen inter-organizational networks among IPs by promoting collaboration aimed at sharing ideas and knowledge construction.

Strengths and Limitations of the study

We obtained a rich and nuanced appreciation of the implementation of the SmartCare as we sampled from a range of IPs, health facilities, stakeholders and implementation systems. The depth of the inquiry enabled us to consider a range of explanatory factors. Most of these factors were related to the EHR system and its implementation. We also considered the factors encountered by the PMTCT clients but these did not emerge as major concerns. This is likely due the fact that the patients do not interact with SmartCare directly.

There are several limitations of the study, such as the restriction to health facilities in and around Lusaka. Lusaka is the capital city of Zambia and therefore the health facilities selected could be receiving financial and technical support of an order that is unlikely to be offered in remote facilities even by the same IP. However, our previous work on the quantitative analysis of PMTCT data does not support this argument as several provinces had better data quality than Lusaka (14, 15).

Another limitation of the study it is that, although we intended to focus on implementation of SmartCare for PMTCT, the issues which arose were related to the entire system. The findings of the study might have been different if stakeholders from other health services such as general ART services were included. The system in Zambia is such that pregnant and lactating women are given 'VIP' treatment which allows them to be served faster under the

For peer review only - http://bmjopen.bmj.com/site/about/guidelines.xhtml

ANC department compared to those under General ART. Despite that the setup offers 'VIP' services, data collection and entry of PMTCT services were noted to be a challenge as in most facilities there is no designated personal for PMTCT data entry. In addition the movement of forms from the PMTCT department to the data entry room was reported to be not well coordinated.

Our study did not investigate how patients relate with the clinicians when they interact with them; although this could have had an impact on both staff and patients' perceptions of SmartCare, it was beyond the scope of our research.

6. Conclusion

The SmartCare system has structural challenges which can be traced from its development. Funding gaps have resulted in staffing and data collection disparities within implementing partners. The lack of feedback from the system has also led to complacency in operational level which has resulted in poor data quality in later years. The data from the database, if appropriately understood, could be used by health facility staff as an advocacy tool, as well as in monitoring the impact of PMTCT programs. Our research could aid other countries wanting to develop their own EHR systems.

(4520 words)

Authors' contributions

Dr Sehlulekile Gumede-Moyo conceived the study and conducted all the interviews and observations under the supervision of Professor Suzanne Filteau, and Mr. Jim Todd. Analysis was conducted by Dr Sehlulekile Gumede-Moyo with the input from Professor Suzanne Filteau, Mr. Jim Todd, Dr Paul Mee and Dr Virginia Bond. The manuscript was drafted by Dr Sehlulekile Gumede-Moyo with contributions from all the authors. All authors read and approved the final manuscript.

Availability of Data

No additional data available.

Competing interests

None declared.

For peer review only

For peer review only - http://bmjopen.bmj.com/site/about/guidelines.xhtml

Acknowledgements

The study was supported by the SEARCH (Sustainable Evaluation through Analysis of Routinely Collected HIV data) Project funded by the Bill & Melinda Gates Foundation grant number OPP1084472. We also thank the Ministry of Health, Department of Policy and Planning for granting us permission to access the SmartCare database. The authors thank all the participants who contributed their time and effort to the study and Dr. Jasleen Singh (MSc student) for going through the transcripts and assessing the initial analysis.

For peer review only

References

1. Gourlay A, Wringe A, Todd J, Michael D, Reniers G, Urassa M, et al. Challenges with routine data sources for PMTCT program monitoring in East Africa: insights from Tanzania. Global health action. 2015;8:29987.
2. Grzeskowiak LE, Gilbert AL, Morrison JL. Methodological challenges in using routinely collected health data to investigate long-term effects of medication use during pregnancy. Ther Adv Drug Saf. 2013;4(1):27-37.
3. Shaw V. Health information system reform in South Africa: developing an essential data set. . Bulletin of the World Health Organization. 2005;83(8):632–6.
4. Mate KS, Bennett B, Mphatswe W, Barker P, Rollins N. Challenges for routine health system data management in a large public program to prevent mother-to-child HIV transmission in South Africa. PloS one. 2009;4(5):e5483.
5. Munthali T. MP, Mee P., Gumede S., Schaap A, Mwinga A., Phiri C., Kapata N., Michelo C., and Todd J. Underutilisation of routinely collected data in the HIV program in Zambia: A review of quantitatively analysed peer-reviewed articles. BMC Research Policy and Systems. 2017;15(51).
6. Gumede-Moyo S, Filteau S, Munthali T, Todd J, Musonda P. Implementation effectiveness of revised (post-2010) World Health Organization guidelines on prevention of mother-to-child transmission of HIV using routinely collected data in sub-Saharan Africa: A systematic literature review. Medicine (Baltimore). 2017;96(40):e8055.
7. Russell A, Hellawell G. The future of electronic health records. Br J Hosp Med (Lond). 2013;74(11):604-5.
8. Namulanda G, Qualters J, Vaidyanathan A, Roberts E, Richardson M, Fraser A, et al. Electronic health record case studies to advance environmental public health tracking. J Biomed Inform. 2018;79:98-104.
9. Cifuentes M, Davis M, Fernald D, Gunn R, Dickinson P, Cohen DJ. Electronic Health Record Challenges, Workarounds, and Solutions Observed in Practices Integrating Behavioral Health and Primary Care. J Am Board Fam Med. 2015;28 Suppl 1:S63-72.
10. Fraser HS, Biondich P, Moodley D, Choi S, Mamlin BW, Szolovits P. Implementing electronic medical record systems in developing countries. Inform Prim Care. 2005;13(2):83-95.
11. Allen C, Jazayeri D, Miranda J, Biondich PG, Mamlin BW, Wolfe BA, et al. Experience in implementing the OpenMRS medical record system to support HIV treatment in Rwanda. Stud Health Technol Inform. 2007;129(Pt 1):382-6.
12. Siika AM, Rotich JK, Simiyu CJ, Kigotho EM, Smith FE, Sidle JE, et al. An electronic medical record system for ambulatory care of HIV-infected patients in Kenya. Int J Med Inform. 2005;74(5):345-55.
13. Oluoch T, Santas X, Kwaro D, Were M, Biondich P, Bailey C, et al. The effect of electronic medical record-based clinical decision support on HIV care in resource-constrained settings: a systematic review. Int J Med Inform. 2012;81(10):e83-92.
14. Singh J, Filteau S, Todd J, Gumede-Moyo S. Progress in the performance of HIV early infant diagnosis services in Zambia using routinely collected data from 2006 to 2016. BMC public health. 2018;18(1):1297.
15. Gumede-Moyo S, Todd J, Schaap A, Mee P, Filteau S. Effect of prevention of mother-to-child transmission strategies on antiretroviral therapy coverage in pregnant women in Zambia: analysis using routinely collected data (2010–15). Lancet Global Health. 2019;7(Supplement 1):S25.
16. Muyunda G. Zambia leads the way in SmartCare electronic health records system, a benefit to both providers and patients 2011 [Available from:

For peer review only - http://bmjopen.bmj.com/site/about/guidelines.xhtml

https://www.jhpiego.org/success-story/zambia-leads-the-way-in-smartcare-electronic-health-records-system-a-benefit-to-both-providers-and-patients/.
17. Lincoln NKDYS. Handbook of Qualitative Research 2nd ed. London: Sage Publications; 2000.
18. Vaismoradi M, Turunen H, Bondas T. Content analysis and thematic analysis: Implications for conducting a qualitative descriptive study. Nurs Health Sci. 2013;15(3):398-405.
19. Graneheim U.H. LB. Qualitative Content Analysis in Nursing Research: Concepts, Procedures and Measures to Achieve Trustworthiness. Nurse Education Today 2004;24:105-12.
20. Abiy R, Gashu K, Asemaw T, Mitiku M, Fekadie B, Abebaw Z, et al. A Comparison of Electronic Medical Record Data to Paper Records in Antiretroviral Therapy Clinic in Ethiopia: What is affecting the Quality of the Data? Online journal of public health informatics. 2018;10(2):e212.
21. Odekunle FF, Odekunle RO, Shankar S. Why sub-Saharan Africa lags in electronic health record adoption and possible strategies to increase its adoption in this region. International journal of health sciences. 2017;11(4):59-64.
22. Fritz F, Tilahun B, Dugas M. Success criteria for electronic medical record implementations in low-resource settings: a systematic review. J Am Med Inform Assoc. 2015;22(2):479-88.
23. Williams F, Boren SA. The role of the electronic medical record (EMR) in care delivery development in developing countries: a systematic review. Inform Prim Care. 2008;16(2):139-45.
24. Ivers NM, Sales A, Colquhoun H, Michie S, Foy R, Francis JJ, et al. No more 'business as usual' with audit and feedback interventions: towards an agenda for a reinvigorated intervention. Implementation science : IS. 2014;9:14.
25. Hemler JR, Hall JD, Cholan RA, Crabtree BF, Damschroder LJ, Solberg LI, et al. Practice Facilitator Strategies for Addressing Electronic Health Record Data Challenges for Quality Improvement: EvidenceNOW. J Am Board Fam Med. 2018;31(3):398-409.
26. Douglas GP, Gadabu OJ, Joukes S, Mumba S, McKay MV, Ben-Smith A, et al. Using touchscreen electronic medical record systems to support and monitor national scale-up of antiretroviral therapy in Malawi. PLoS medicine. 2010;7(8).
27. Greenhalgh T, Robert G, Macfarlane F, Bate P, Kyriakidou O. Diffusion of innovations in service organizations: systematic review and recommendations. The Milbank quarterly. 2004;82(4):581-629.

For peer review only

For peer review only - http://bmjopen.bmj.com/site/about/guidelines.xhtml

Table1: Breakdown of In-depth Interviews and Focus Group Discussions Participants

In-depth interviews with key informants	
Leadership	2 Implementing partners, 1 Ministry of Health representative
Database management	1 Ministry of Health ICT Officer, 2 Data Managers, 2 Monitoring and Evaluation Officers, 1 Database Developer
Data Entry Clerks	1 computer-based health facility, 4 paper-based health facilities
MCH- Nurse-in charge	1 computer –based health facility, 2 paper-based health facility
**Focus group discussions with service users**	
Peri-urban health facility	4 pregnant, 2 lactating women
Paper- based health facility	4 pregnant, 4 lactating women
Computer-based health facility	4 pregnant, 4 lactating women

For peer review only

Figure Legends

Figure 1

The Ministry of Health of Republic of Zambia is the copyright holder of the figure

Figure 1 Abbreviations

ANC	Antenatal Care
EHR	Electronic Health Records
HMIS	Health Management Information System
HIA	Health Information Aggregation
TB	Tuberculosis
OPD	Out Patient Department

Figure 2

The Ministry of Health of Republic of Zambia is the copyright holder of the figure

For peer review only

For peer review only - http://bmjopen.bmj.com/site/about/guidelines.xhtml

Figure 1: SmartCare Model Structure

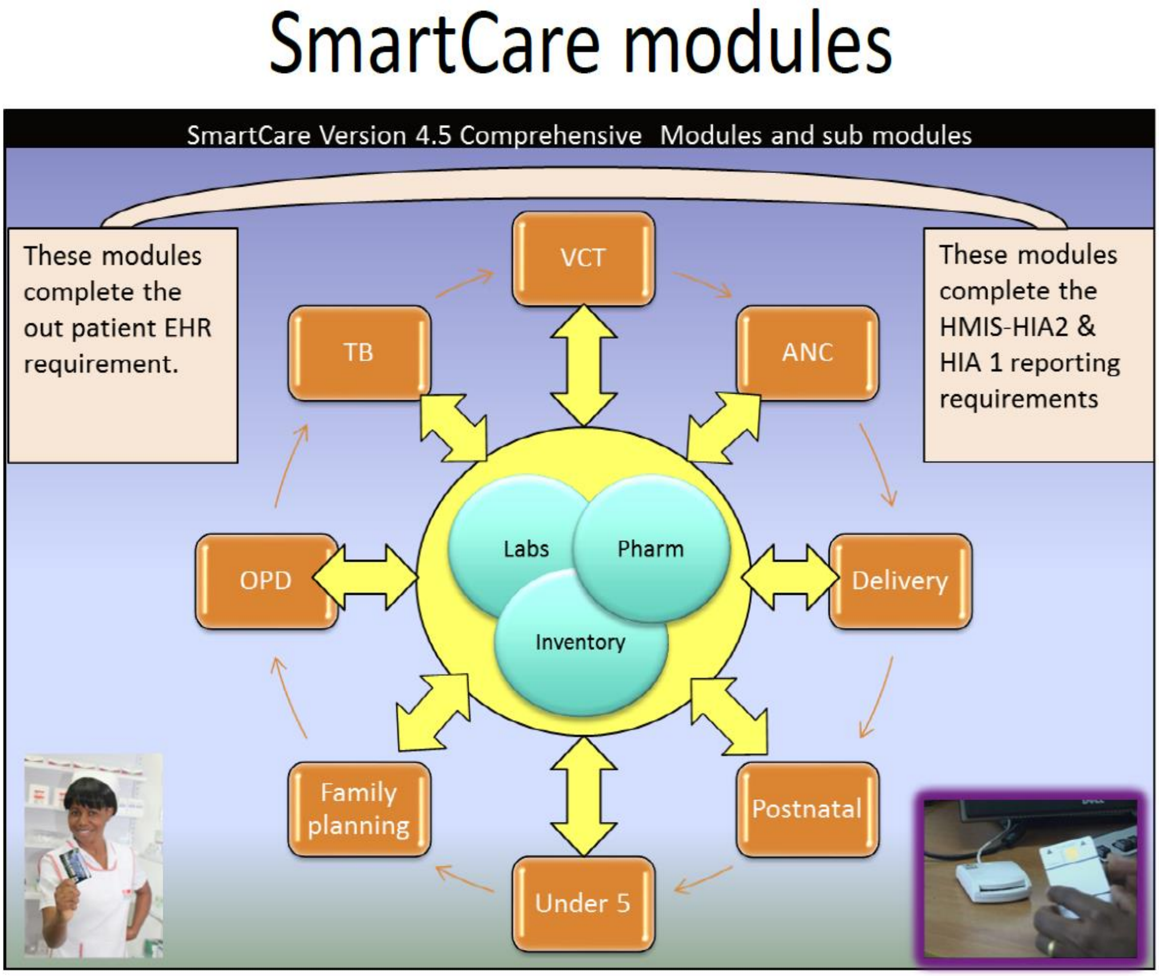

Adapted from Ministry Health HMIS workshop Presentation 2016

Figure 2: SmartCare Data flow

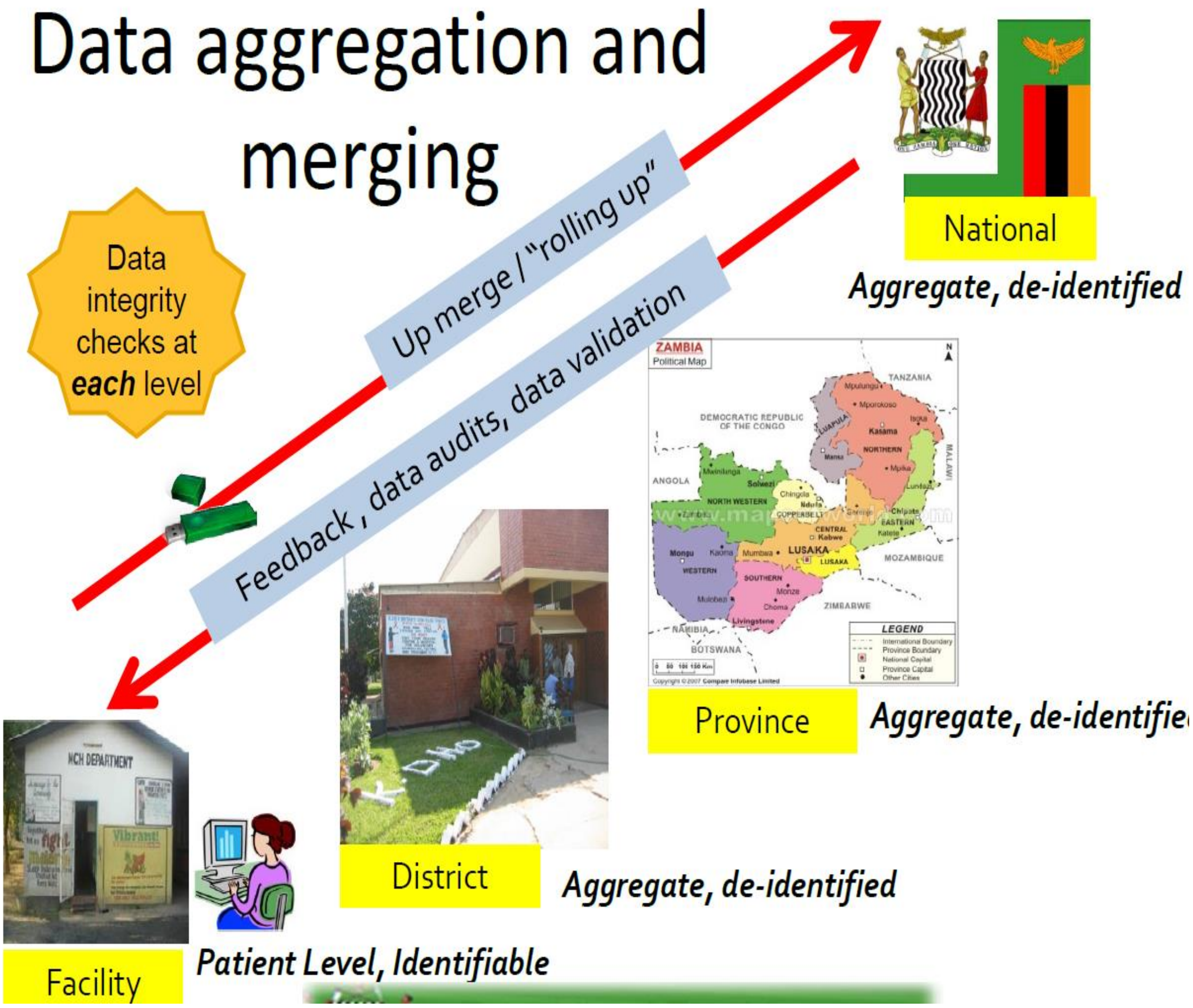

Adapted from Ministry Health HMIS workshop Presentation 2016

only

COREQ (COnsolidated criteria for REporting Qualitative research) Checklist

A checklist of items that should be included in reports of qualitative research. You must report the page number in your manuscript where you consider each of the items listed in this checklist. If you have not included this information, either revise your manuscript accordingly before submitting or note N/A.

Topic	Item No.	Guide Questions/Description	Reported on Page No.
**Domain 1: Research team and reflexivity**			
Personal characteristics			
Interviewer/facilitator	1	Which author/s conducted the interview or focus group?	7
Credentials	2	What were the researcher's credentials? E.g. PhD, MD	1
Occupation	3	What was their occupation at the time of the study?	1
Gender	4	Was the researcher male or female?	N/A
Experience and training	5	What experience or training did the researcher have?	1
Relationship with participants			
Relationship established	6	Was a relationship established prior to study commencement?	4
Participant knowledge of the interviewer	7	What did the participants know about the researcher? e.g. personal goals, reasons for doing the research	6
Interviewer characteristics	8	What characteristics were reported about the inter viewer/facilitator? e.g. Bias, assumptions, reasons and interests in the research topic	6-7
**Domain 2: Study design**			
Theoretical framework			
Methodological orientation and Theory	9	What methodological orientation was stated to underpin the study? e.g. grounded theory, discourse analysis, ethnography, phenomenology, content analysis	5
Participant selection			
Sampling	10	How were participants selected? e.g. purposive, convenience, consecutive, snowball	6-7
Method of approach	11	How were participants approached? e.g. face-to-face, telephone, mail, email	7
Sample size	12	How many participants were in the study?	
Non-participation	13	How many people refused to participate or dropped out? Reasons?	6
Setting			
Setting of data collection	14	Where was the data collected? e.g. home, clinic, workplace	7
Presence of non-participants	15	Was anyone else present besides the participants and researchers?	7-8
Description of sample	16	What are the important characteristics of the sample? e.g. demographic data, date	6 & 7
Data collection			
Interview guide	17	Were questions, prompts, guides provided by the authors? Was it pilot tested?	7
Repeat interviews	18	Were repeat inter views carried out? If yes, how many?	17
Audio/visual recording	19	Did the research use audio or visual recording to collect the data?	7-8
Field notes	20	Were field notes made during and/or after the inter view or focus group?	8
Duration	21	What was the duration of the inter views or focus group?	7
Data saturation	22	Was data saturation discussed?	N/A
Transcripts returned	23	Were transcripts returned to participants for comment and/or	N/A

For peer review only

Topic	Item No.	Guide Questions/Description	Reported on Page No.
		correction?	
**Domain 3: analysis and findings**			
Data analysis			
Number of data coders	24	How many data coders coded the data?	8
Description of the coding tree	25	Did authors provide a description of the coding tree?	9
Derivation of themes	26	Were themes identified in advance or derived from the data?	8
Software	27	What software, if applicable, was used to manage the data?	8
Participant checking	28	Did participants provide feedback on the findings?	N/A
Reporting			
Quotations presented	29	Were participant quotations presented to illustrate the themes/findings? Was each quotation identified? e.g. participant number	8
Data and findings consistent	30	Was there consistency between the data presented and the findings?	N/A
Clarity of major themes	31	Were major themes clearly presented in the findings?	9-15
Clarity of minor themes	32	Is there a description of diverse cases or discussion of minor themes?	15-17

Developed from: Tong A, Sainsbury P, Craig J. Consolidated criteria for reporting qualitative research (COREQ): a 32-item checklist for interviews and focus groups. *International Journal for Quality in Health Care*. 2007. Volume 19, Number 6: pp. 349 – 357

Once you have completed this checklist, please save a copy and upload it as part of your submission. DO NOT include this checklist as part of the main manuscript document. It must be uploaded as a separate file.

For peer review only

BMJ Open

A qualitative inquiry into implementing an electronic health record system (SmartCare) for prevention of mother-to-child transmission data in Zambia- A retrospective study.

Journal:	*BMJ Open*
Manuscript ID	bmjopen-2019-030428.R2
Article Type:	Research
Date Submitted by the Author:	06-Aug-2019
Complete List of Authors:	Gumede-Moyo, Sehlulekile; London School of Hygiene and Tropical Medicine, Epidemiology and Population Health<br>Todd, Jim; London School of Hygiene and Tropical Medicine Faculty of Epidemiology and Population Health<br>Bond, Virginia; London School of Hygiene and Tropical Medicine Faculty of Epidemiology and Population Health<br>Mee, Paul; London School of Hygiene and Tropical Medicine Faculty of Epidemiology and Population Health<br>Filteau, Suzanne; London School of Hygiene and Tropical Medicine Faculty of Epidemiology and Population Health
**Primary Subject Heading**:	Health informatics
Secondary Subject Heading:	Health services research, Qualitative research
Keywords:	PMTCT, routinely collected data, electronic health records

SCHOLARONE™
Manuscripts

A qualitative inquiry into implementing an electronic health record system (SmartCare) for prevention of mother-to-child transmission data in Zambia- A retrospective study.

Sehlulekile Gumede-Moyo (BSc, MPH, MBA, PhD,)[1,2§]; Jim Todd (MA, MSc)[1] ; Virginia Bond (MSc, PhD)[1,3] Paul Mee (BSc, MSc, PhD)[1] and Suzanne Filteau (BSc, MSc, PhD)[1]

1. Department of Population Health, Faculty of Epidemiology and Population Health, London School of Hygiene and Tropical Medicine, London, UK

2. School of Public Health, University of Zambia, Lusaka, Zambia

3. ZAMBART, University of Zambia, School of Medicine

[§]Corresponding author: Sehlulekile Gumede-Moyo. Department of Population Health, Faculty of Epidemiology and Population Health, London School of Hygiene and Tropical Medicine, Keppel Street, London WC1E 7HT, UK. (sehlulekile.gumede@lshtm.ac.uk)

Email addresses of authors:

SGM: sehlulekile.gumede@lshtm.ac.uk

JT: jim.todd@lshtm.ac.uk

VB: virginia.bond@lshtm.ac.uk

PM: paul.mee@ lshtm.ac.uk

SF: suzanne.filteau@lshtm.ac.uk

For peer review only

Key words: PMTCT, routinely collected data, electronic health records, implementation.

Abstract

Objective: This study aimed to investigate the challenges in implementing a Zambian Electronic Health Records (EHR) system labelled 'SmartCare' from diverse stakeholder perspectives in order to improve PMTCT data collection so that SmartCare can be used for clinic performance strengthening and program monitoring.

Design: This qualitative retrospective study.

Setting and participants: SmartCare is a Zambian Ministry of Health (MoH)-led project funded by the United States Centre for Disease Control and Prevention (CDC). Data were collected using in-depth interviews, observations and focus groups discussions (FGDs) between September and November 2016. Seventeen in-depth interviews were held with a range of key informants from the Ministry of Health and local and international organisations implementing SmartCare. Four data entry observations and three FGDs with 22 pregnant and lactating women seeking PMTCT services were conducted. Data were analysed using a thematic content approach.

Results: The SmartCare system has evolved from various patient tracking systems into a multifunctional system. There is a burden of information required so that sometimes not all is collected and entered into the database, resulting in poor data quality. Funding challenges impede data collection due to manpower constraints and shortages of supplies. Challenges associated with data collection depend on whether a paper- or computer-based system is utilised. There is no uniformity in the data quality verification and submission strategies employed by various implementing partners. There is little feedback from the EHR system at health facility level, which has led to disengagement as stakeholders do not see the importance of the system.

Conclusion: SmartCare has structural challenges which can be traced from its development. Funding gaps have resulted in staffing and data collection disparities within implementing partners. The lack of feedback from the system has also led to complacency at the operational level which has resulted in poor data quality in later years.

(297 words)

For peer review only - http://bmjopen.bmj.com/site/about/guidelines.xhtml

Strengths

- We obtained a rich and nuanced appreciation of the implementation of the SmartCare as we sampled from a range of IPs, health facilities, stakeholders and implementation systems.
- The depth of the inquiry enabled us to consider a range of explanatory factors.

Weaknesses

- Data collection was restricted to health facilities in and around Lusaka, the capital city of Zambia, and therefore the health facilities selected could be receiving financial and technical support of an order that is unlikely to be offered in remote facilities.

For peer review only

For peer review only - http://bmjopen.bmj.com/site/about/guidelines.xhtml

Introduction

Routinely collected clinic data can provide much needed information on the prevalence of HIV among pregnant women and the uptake of services for prevention of mother-to-child transmission (PMTCT) of HIV(1). The use of routinely collected data can be timely and cost-efficient for decision making as data are already available for analysis (2). Collection of high-quality routine data on these services and outcomes for HIV-positive mothers and HIV-exposed infants is essential for monitoring and evaluation of PMTCT programs, for clinical management of patients, and for managing stocks of HIV test kits and drugs (1). For both clinic staff and health system managers, having access to reliable data that reflects the processes of care and clinical outcomes is the first step to ensuring effective delivery of an intervention within a health care system (3, 4).However in Zambia there is underutilization of routinely collected data in the HIV programs (5).

Data collection systems for PMTCT programs in Africa often lack immediacy as many are paper-based with records completed at each facility, and with individual level data that are inaccessible to central planners (6). Electronic health records (EHRs) could present an opportunity to supplement current sources of routinely collected surveillance data (7). EHRs are real-time, patient-centred records that make information available instantly and securely to authorized users (8). While an EHR does contain the medical and treatment histories of patients, an EHR system is built to go beyond standard clinical data collected in a provider's office and can include a broader view of a patient's care (9).

Various EHR systems are implemented globally (10-12). A systematic of review of literature about EHR systems in resource-constrained settings recommended that it is urgent to evaluate barriers to implementation (13). Our own research on extracting surveillance data from a Zambian EHR system, SmartCare, has highlighted some deficiencies including large amounts of missing data, especially in more recent years, and variable performance across the country (14, 15). This study aimed to investigate the challenges in implementing SmartCare from diverse stakeholder perspectives in order to improve PMTCT data collection so that it can be used for clinic performance strengthening and program monitoring. For the context of our study, implementation of SmartCare was based on getting good data from individuals attending health facilities. The later stages involving retraining of staff and reorganizing supply chains are not included in this definition of implementation.

For peer review only - http://bmjopen.bmj.com/site/about/guidelines.xhtml

Methods

Study design

This qualitative retrospective study included in-depth interviews, observations and focus groups discussions (FGDs) conducted between September and November 2016.

Study Setting

SmartCare is a Zambian Ministry of Health (MoH)-led project funded by the United States Centre for Disease Control and Prevention (CDC). SmartCare was developed to improve continuity of care and provide timely data on maternal and child health, HIV/AIDS, tuberculosis and malaria interventions for public health purposes, trend reporting and analysis for health officials and clinicians (16). The Implementing partners (IPs) are responsible for data entry mainly from paper-based forms which have information collected by clinicians which is then entered into a computer.

The SmartCare database is a derivative of the PTS (patient tracking system) which was developed in 2004, based on a health facility-centred EHR system. In 2010, it became a national medical health program and was then rolled out throughout the country. It is implemented by government, and both international and local organisations primarily for patient management. Most of the international organisations were involved in the database development, and the local organisations became involved when implementation was rolled out throughout the country (after 2009).

SmartCare is organised into comprehensive modules and sub-modules ***(Figure 1).*** This was mainly influenced by its funders depending on the information they wanted at that particular time. The main module groups are: clinical group for which the modules of interest in this study are Antenatal care(ANC), Delivery, Postnatal, Antiretroviral therapy (ART), Paediatric ART, PMTCT follow up, and Under 5; logistic group which has information on drug dispensing and orders; monitoring and evaluation groups which includes health management information system reports, graphing, data analysis, data merge from facilities for MOH; and the continuity of care group which has data from across facilities and within facilities. Through the data-merge the SmartCare information can be used to obtain data for the monthly reports to the MOH.

For peer review only - http://bmjopen.bmj.com/site/about/guidelines.xhtml

Data are captured under a number of 'modules' across a range of health issues. Records are updated at every point of clinical service. Patients are issued with Smartcards at their initial consultation which contains all their clinical information and treatment details and can be accessed from any SmartCare facility.

The SmartCare data are collected either directly onto a computer or using a paper-based method. When using the computer-based data collection and entry method, clinicians have to enter data directly in the computer; this method is still being piloted in a few facilities mainly in the Southern and Lusaka provinces. For the more common paper-based data method, client information is documented in the SmartCare care forms. The papers are then taken to the data entry room where the information is entered into the computer. Some facilities which are deep rural (where there is no electricity at all) use paper only and the forms are then transferred to a location where there are computers and entered into the database.

Data on a range of HIV and pregnancy-related outcomes are collected during the patient consultation with the clinician, who records the information on paper forms or directly on the computer in the few facilities which are computer-based. The paper forms are then entered into the SmartCare database by data entry clerks who have been trained in using SmartCare. Data are collected from each facility on a monthly basis and submitted to a District Health Information Officer, who aggregates and sends the data to the province level Senior Health Information Officers. From here the data are wired to a national server at MoH headquarters ***(Figure 2).***

For the purposes of this study, routine data are defined as data that are routinely generated through ANC and PMTCT service delivery, and routinely recorded in standard SmartCare data tools.

Sampling

Data were generated from 17 in-depth semi-structured face-to-face interviews, four data entry observations and three FGDs with 22 pregnant and lactating women seeking PMTCT services from three health facilities (Table 1).

Key informant interviews

The IPs were purposively sampled in consultation with the MoH to identify diverse organizations. Six health facilities were selected based on local considerations which

For peer review only - http://bmjopen.bmj.com/site/about/guidelines.xhtml

included: both well-resourced international organizations and less well-resourced local and government-owned organizations; those best performing versus struggling; those which are paper- or computer-based; and facilities which had already transitioned to 'test and treat' for general population HIV care. Most of the facilities sampled were in Lusaka City, and only one was peri-urban. The choice of Lusaka was based on the following considerations: 1) logistics, 2) facilities in Lusaka are busy and have the highest case loads, 3) our previous work has shown that facilities in Lusaka show a wide range of successes and failures, and 4) overall Lusaka performs poorly compared to the rest of the country and therefore it was important to investigate the working of SmartCare in Lusaka city.

Within each of the selected IPs, a diverse range of interviewees were purposively recruited in order to give the broadest range of perspectives (17, 18). The approaches to recruitment of participants were flexible, being negotiated with key contacts and recommendations from managers who were also considered for in-depth interviews. Interviews with key informants were conducted by the first author in English. They lasted 30 to 60 minutes and consisted of a series of open-ended questions with follow-up question probes. The interview guides were specific to the hierarchical levels of operation, namely: leadership level (persons involved in the strategic direction of SmartCare); operational level (Data Managers and Monitoring and Evaluation personnel); and implementation level (Data entry clerks and MCH in-charge clinicians). The interview guides were designed to capture information on SmartCare development, implementation procedures, data quality management and linkage between SmartCare and PMTCT services.

Focus group discussions

The FGDs were conducted at maternal and child health (MCH) departments with participants from three different facilities, two from Lusaka and one from the peri-urban area. The participants were recruited with the assistance of PMTCT coordinators who were instructed to balance the number of pregnant and lactating women on ART. The FGD sessions were conducted by the first author and a co-moderator who was fluent in the local languages (Bemba and Nyanja). These discussions were guided by an interview guide containing open-ended questions and lasted for 60 to 90 minutes. The interview guide was designed to capture the general knowledge and experience of the PMTCT program and SmartCare operations.

For peer review only

For peer review only - http://bmjopen.bmj.com/site/about/guidelines.xhtml

Facility observations

The observations were conducted to familiarise the researcher with the realities of the SmartCare data collection and entry environments. These were planned to be conducted in 6 health facilities through interaction with study participants and relevant actors as well as personal reactions to the related events without the use of a specific tool. However in 2 health facilities data entry was not taking place on the scheduled visit days due to power outage, hence only in-depth interviews were conducted with implementing level staff.

2.5 Data Management and Analysis

The in-depth interviews and FGDs were tape-recorded and subsequently transcribed verbatim. FGDs were then translated into English. Data were collected aiming to get sufficient data from relevant actors/perspectives to ensure it was qualitatively representative(19). Detailed field notes were taken on the sites following the observations for analysis.

A thematic content approach was conducted; this was done by reading the transcribed material systematically, line by line, in order to identify the meaning units. Meaning units were defined as strings of the text expressing a single coherent thought, up to the point at which the coherent thought changed. Thereafter, the meaning units were marked by a code, describing what the text unit was about. Coding was also done on issues that were related to the reasons for missing PMTCT data; text unrelated to these issues was not included in the present analysis.

The codes were defined and organised so that those referring to the same subject were grouped into categories. The interview guide was used as a starting point for grouping the information, but during the analysis new categories were developed. When this happened, we re-coded. The data were managed manually in Excel sheets. The underlying meanings of the categories were formulated into a theme. The original data were re-assessed by an assistant (MSc Student) after analysis in order to detect any concepts or information that would have been missed. The first author discussed the results with SF, JT and the MSc student.

3. Research ethics

Written informed consent was obtained from all key informants including their consent to record the interviews and publish anonymous quotations. Verbal consent was sought for FGD participants. Participation in the study was entirely voluntary and participant anonymity was maintained throughout the processes of interview transcription, data analysis and presentation

For peer review only - http://bmjopen.bmj.com/site/about/guidelines.xhtml

by using pseudonyms. Permission to collect data from the health facilities was granted by the MoH district offices. The study was approved by the Zambia Biomedical Ethical Board (Ref 101-04-16) and the LSHTM Research Ethics Committee (Ref 12086).

Patient and public involvement

Patients were not involved in the development of the research question, design of the study and conduct of the study. The findings will be shared with the policy makers in order to help them to improve the implementation of the EHR system.

4. Results

The comments from key informants, observations and the focus group discussions enabled us to characterise four fundamental issues that are key in the implementation of SmartCare: database development and ownership, funding and staffing, the data collection process, and health facility set up.

4.1 Database Development and Ownership

The development of SmartCare was mainly CDC-led, with the government of Zambia through the MoH supporting the process. Multiple stakeholders using various patient tracking software were brought together to form SmartCare. Reflecting on the history of SmartCare, two IP informants identified the leading role of CDC and the subsequent collaboration with a broader group of partners.

'The initial development was by the American-funded institutions and a few people from the MoH because they had gone into partnership. They were developers at MoH and also at CDC, the rest of the institutions like us were only required to use the software.'- Implementing partner leader.

'There were other systems that were used by other partners throughout the country e.g., there was a system called the CareWare which was similar to SmartCare but it was just a patient summary. Then there was another system which was used in the private sector which l can't remember the name. Lessons were drawn from all these systems and brought into one and this is how SmartCare was born.'- Implementing partner leader.

For peer review only - http://bmjopen.bmj.com/site/about/guidelines.xhtml

For peer review only

There was a consensus among the leaders of the SmartCare implementation that the database has also evolved from a system to track patients to its current form as a mixture of a patient tracking tool, a clinical care tool, a reporting tool, and a surveillance tool.

'...it was just a mixture of ideas which were clinically required but each person had different needs for information, some needed it for research purposes, l needed it for patient follow up, another person needed it for forecasting and quantification.'- Implementing partner leader.

The different stakeholders had different data needs which took the form of added modules. The changes have however, made it difficult to classify and use the database because it now tries to address multiple issues simultaneously.

*'I wanted my things as well and partly l wanted things that PEPFAR (*The United States President's Emergency Plan for AIDS Relief) *wanted because our money comes from them. If I can't report on PEPFAR indicators, l don't get more money.' – Implementing partner leader.*

The resulting SmartCare forms are 6-7 pages for a normal interaction per visit because of requirements from its various stakeholders. In addition, a lot of data has been entered into the system making it bulky. This has caused the system to become very slow, and in the mornings it takes a very long time to reboot as narrated by operational and implementing level participants.

'Because the system is slow they have to wait instead of entering 100 files per day they are only able to enter 60 or so.'- Database Manager.

Data entry is often interrupted as the system also hangs up every now then because it is so bulky. Hence a lot of backlogs are experienced which results in a lot of data not being entered.

'Last year it had serious faults and we were not using it for a while.' – Data Entry Clerk.

Although there is a SmartCare technical working group, chaired by the MoH where operations are discussed, it is not clear who is guiding the process, and how the database should be modified to make it work better.

For peer review only - http://bmjopen.bmj.com/site/about/guidelines.xhtml

4.2 Funding and staffing

The database operations are managed by the monitoring and evaluation (M&E) departments of the IPs. The structures of the M&E departments differ across the IPs, with international organisations having much bigger and better resourced departments. The local IPs have much leaner structure, rely mostly on volunteers, and run into problems due to financial constraints. This was elaborated by the leadership and implementing level participants.

'Mostly the people who enter data in the health facilities where we operate are employed by us. Apart from that we also provide support through volunteers; in most cases we also provide peers and counsellors.' – Data Base Manager.

The manpower constraint in the local partners has resulted in the loss of data during long grant negotiations. Data are lost as data entry staff are laid off between funding cycles since their salaries are dependent on grants. In addition, these partners have designated personnel who enter general ART data whilst PMTCT data are mainly entered when the data entry personnel have specific interest or have trained temporary volunteer staff.

'The positions of data entry clerks in the facilities are actually paid for by the donor. It's not sustainable because the moment the donor says we are pulling out, there will not be any data entry personnel in these facilities.'- Implementing partner leader.

The funding of operations also affects the supplies required for the day-to-day operations as narrated by both operations and implementing personnel.

'We normally run out of forms, especially when funding is out. When we don't have funding the facilities also have challenges in printing the forms because toner is expensive.'- Data Entry Clerk.

'….when the computers are down because of the virus, data entry doesn't happen.'- M &E Officer.

In some small facilities where the MoH is solely responsible for data collection and entry, there are no data entry clerks and hence clinicians enter the data on the computer after hours. This is usually done only by staff that are passionate and so give it time according to a MoH leader.

'In the government facilities there is no designated person to manage ART data for SmartCare; its health workers who just do that on their own.' - Implementing partner leader.

For peer review only - http://bmjopen.bmj.com/site/about/guidelines.xhtml

4.3 Data Collection Process

4.3.1 Data entry

With both the computer and the paper-based methods the greatest challenge is incomplete forms which translate to missing data in the database. In some facilities which are computer-based, the data entry clerks noticed that there are also some clinicians who still prefer entering data on paper and handing over to the data entry clerks to enter on the computer.

'In as much as data are supposed to be entered at each service point, some people prefer entering the data on paper and they hand over the data to me.'- Data Entry Clerk.

According to implementing level staff, with the paper-based method, once the client leaves the facility it is a challenge to follow up on the missing data on the forms.

'There are usually many gaps in the forms; the forms are not fully completed more than half of the time.'- Data Entry Clerk.

'When the forms are not complete l return them to whoever was supposed to be entering the data at that service point but the challenge is that they will tell you the person has gone and that can't follow them up and even so in the next visit they will not even bother correcting the information.'- Data Entry Clerk.

One of the MoH participants explained that the incomplete data forms could be a result of the competing demands to physically attend to clients and fill out the forms in the high volume facilities.

'.... It takes 20-30 minutes just to enter the data for one interaction. In clinics where there are many patients, how many patients are you going to see in a day?' – Implementing partner leader.

The implementation challenges of the SmartCare database depend on the data collection and entry method used. Power outages were the greatest challenge: under the computer-based method, client information is not collected, as some clinicians will be reluctant to use the alternative method, whereas with the paper-based method there will be a backlog of work.

Some well-funded partners have installed solar power systems in the health facilities, and this enables them to enter data without interruptions. Despite having good power supply, there is

For peer review only - http://bmjopen.bmj.com/site/about/guidelines.xhtml

a challenge in these facilities of some clinicians who are not computer literate. Furthermore, because of other competing health facility priorities, the power is rarely used for data entry.

'......would you want to power theatre activities or you want to power computers for data entry? Theatre is life saving and data can be entered anytime.' – Implementing partner leader.

4.3.2 Data quality verification

Verification of data quality is specific to each implementing partner. The verification process involves comparing the patient information on the paper forms and registers against the information in the database. The international partners also have verification procedures imbedded in the Monitoring and Evaluation strategies as narrated by one of their participants.

'We are doing double data entry for 10% of our files.' – Data Manager.

On the other hand, verification is rarely done by the local organisation due to manpower constraints as indicated by an operational level participant.

'When l get time, l check what is in the system versus what is in the registers. However it's rare that l get that time because l always have files to enter into the system. In that case l wait for the Quality Control people from Head Office to come and do random verifications for me.'- Data entry clerk.

All the partners reported that there are some instances where they can't find patient files, making it difficult for them to verify the data that is in the computer with the raw data.

'There are certain instances where we can't find patient files.' – M&E Officer.

The patient files will tell me that a facility has 200 clients whilst SmartCare has 300 clients; this is because some of these files are kept somewhere, could be files of staff members or people with prominent positions in that particular area.' – Database Manager.

The SmartCare Database system has reports that are built in the application. However all the partners alluded that these reports are very inconsistent and incorrect.

'In some cases it will show a large number of people who would have been initiated in a day more than what we would have actually done.' – MCH Nurse in Charge.

For peer review only - http://bmjopen.bmj.com/site/about/guidelines.xhtml

4.3.3. Submission of data to the main database

There is also no uniformity in submission of the data to the main database, although according to the MOH the data are supposed to flow from the facility to the district, province and then to the main database at head office ***(Figure 2).***

'They sometimes come to the facilities and collect the data on their own since they are the ones who own these facilities as well.' – M & E officer.

Some partners wait for MoH to request data whilst some submit their data to CDC.

'We do not follow that procedure as per say because we collect data directly from the sites and aggregate it from here and submit to CDC. If MOH requests it, we submit to them but we are not that consistent.'- Database Manager.

4.3.4Feedback from Database

There is no standardised procedure for reporting data that is collected by IPs. The partners normally use the information internally as well as sharing with the donors supporting them.

'We also have special reports that we do in a monthly basis such as the PEPFAR report.' – M & E Coordinator.

In contrast, the nurses-in-charge in some of the facilities reported that they have never received feedback from the system.

'We have not received anything; they don't give us any reports. We would, however, want to receive reports so that we would know how we are faring.' – MCH Nurse in Charge.

As viewed by one of the leaders who participated in the study, the lack of feedback from the system has led to disengagement and some stakeholders not seeing the importance of documenting client information in the SmartCare Database.

'*A lot of the staff collecting this data do not understand how and why they are collecting this data and they are frustrated by it as it also takes lots of their time entering this data' – Implementing partner leader.*

4.4 Health Facility setup

The setup in some health facilities is in such a way that the PMTCT services are offered in the ANC department whereas ART services are offered in places designated solely for ART.

For peer review only - http://bmjopen.bmj.com/site/about/guidelines.xhtml

The nurses in charge in such health facilities narrated that the facility setup could be a great challenge in ensuring that the PMTCT data are entered in an efficient manner.

'Probably if it was housed within the ART department, you could have the same person entering the PMTCT data into the SmartCare.' – MCH nurse in charge.

In addition, the MCH nurses-in-charge indicated that client files sometimes go missing between the MCH and the data room.

'I am sure when they are sending the files; some are lost during transfer in different departments.' – MCH nurse-in-charge.

For peer review only

Views of pregnant and lactating women

Overall, women seeking PMTCT services did not have concerns on the implementation of SmartCare, as according to them the healthcare workers will be doing their job.

'The person who is supposed to enter the data does so because he was trained anyway.'- PMTCT FGD Mothers.

'We just carry our cards and leave them to do their job.'- PMTCT FGD Mothers.

'We are here for our lives and that of our babies, we have to comply and be patient.'- PMTCT FGD Mothers.

5. Discussion

The study has provided evidence on the SmartCare implementation challenges. It has also provided insights as to how to improve implementation, and data quality. The main points were to collect less, but better data, to engage the clinic staff by providing regular feedback, and to improve the software. Fortunately, PMTCT clients appear already satisfied with the system.

The design of EHR systems does not always get attention due to pressures related to their functionality requirements (10). SmartCare was exposed to pressures as its data model evolved from a patient tracking system into a multi-functional system due to demands of various stakeholders. In an effort to shape the SmartCare database into a manageable tool that

For peer review only - http://bmjopen.bmj.com/site/about/guidelines.xhtml

can be used for public health purposes, CDC and MOH could streamline the process for including and excluding information that is collected. There is a need to cut down on the number of fields collected so that less data of higher quality are collected. This could improve the system by: reducing the amount of data so that it takes less time to collect and people might more readily do it; and having a less bulky database which will be less likely to be affected by computer crashes and faster to use.

SmartCare could be developed into a networked EHR, which has been reported to be successful in other developing countries like Rwanda, and Kenya (20). In a networked EHR data can be accessible and be shared in multiple sites; multiple users can enter data simultaneously; data can be backed up automatically at more than one site; information can be communicated between multiple locations; data are accessible and shared at multiple sites and the system can be debugged and upgraded over the internet without visiting remote sites (10, 21). This would allow greater use of the data by health workers and Ministry planners. However, it is important that there is uniformity in the implementation of SmartCare throughout the country so that everyone will be on the same level. This will allow for comparisons across geographic regions and longitudinal analyses.

In Ethiopia the quality of data was reported to be affected by dual documentation where both paper based and electronic systems were used (22). In this study completeness of paper-based records was slightly better than electronic records. According to Odekunle et al limited computer skills is one of the factors affecting electronic health record adoption in sub-Saharan Africa. The authors pointed out that in some countries, many physicians and other key end users are not eager to adopt an EHR, resulting in low EHR adoption in the region (23).

There was a notable lack of appreciation of the system, and a need to train and support end users of the system such as the clinicians who are directly involved in the data collection process. For both the clinic staff and health system managers, having access to reliable data that reflects the processes of care and clinical outcomes is the first step to ensuring effective delivery of an intervention within a health care system(4). Hence, there should be better buy-in of the staff at the facilities in order to make sure that they fill in the forms regularly and accurately.

For peer review only

For peer review only - http://bmjopen.bmj.com/site/about/guidelines.xhtml

In our findings workload affected the documentation, as evidenced by poor data quality in the quantitative analysis of SmartCare PMTCT data (14, 15). In low–resourced countries there is a shortage of a qualified work-force and most health care institutions do not have dedicated IT staff for their EHR systems (24). The results of our study are consistent with a systematic review of literature on the role of EHR systems in developing countries which pointed to the lack of financial and human resources as major challenges in implementing EHR systems (20). Therefore, there is a great need for strong commitment from both the government and the donors to invest in improving the implementation of the system.

The data from the database should be presented to facility staff as an advocacy tool which is needed in their facilities, so that they would appreciate the need for it (21, 25). Similarly, in their description of the rationale and experience in scaling up EHR in Malawi, Douglas and colleagues concluded that health workers will not adopt a system if they do not find sufficient value in it (26). It is also possible that health workers perceive SmartCare as complex, and of little value to their work; therefore any revision to the system would require a clear strategy for ensuring that the operations are well conceptualized (27).

The knowledge required for the use of the database should easily be transferred through training, ensuring that it is customized to the IPs' requirements and the establishment of helpdesk call centres to readily assist with operational challenges. There is also a need to strengthen inter-organizational networks among IPs by promoting collaboration aimed at sharing ideas and knowledge construction.

Strengths and Limitations of the study

We obtained a rich and nuanced appreciation of the implementation of the SmartCare as we sampled from a range of IPs, health facilities, stakeholders and implementation systems. The depth of the inquiry enabled us to consider a range of explanatory factors. Most of these factors were related to the EHR system and its implementation. We also considered the factors encountered by the PMTCT clients but these did not emerge as major concerns. This is likely due the fact that the patients do not interact with SmartCare directly.

There are several limitations of the study, such as the restriction to health facilities in and around Lusaka. Lusaka is the capital city of Zambia and therefore the health facilities selected could be receiving financial and technical support of an order that is unlikely to be

For peer review only - http://bmjopen.bmj.com/site/about/guidelines.xhtml

offered in remote facilities even by the same IP. However, our previous work on the quantitative analysis of PMTCT data does not support this argument as several provinces had better data quality than Lusaka (14, 15).

Another limitation of the study it is that, although we intended to focus on implementation of SmartCare for PMTCT, the issues which arose were related to the entire system. The findings of the study might have been different if stakeholders from other health services such as general ART services were included. The system in Zambia is such that pregnant and lactating women are given 'VIP' treatment which allows them to be served faster under the ANC department compared to those under General ART. Despite that the setup offers 'VIP' services, data collection and entry of PMTCT services were noted to be a challenge as in most facilities there is no designated personal for PMTCT data entry. In addition the movement of forms from the PMTCT department to the data entry room was reported to be not well coordinated.

Our study did not investigate how patients relate with the clinicians when they interact with them; although this could have had an impact on both staff and patients' perceptions of SmartCare, it was beyond the scope of our research.

6. Conclusion

The SmartCare system has structural challenges which can be traced from its development. Funding gaps have resulted in staffing and data collection disparities within implementing partners. The lack of feedback from the system has also led to complacency in operational level which has resulted in poor data quality in later years. The data from the database, if appropriately understood, could be used by health facility staff as an advocacy tool, as well as in monitoring the impact of PMTCT programs. Our research could aid other countries wanting to develop their own EHR systems.

(4520 words)

For peer review only

For peer review only - http://bmjopen.bmj.com/site/about/guidelines.xhtml

Authors' contributions

Dr Sehlulekile Gumede-Moyo conceived the study and conducted all the interviews and observations under the supervision of Professor Suzanne Filteau, and Mr. Jim Todd. Analysis was conducted by Dr Sehlulekile Gumede-Moyo with the input from Professor Suzanne Filteau, Mr. Jim Todd, Dr Paul Mee and Dr Virginia Bond. The manuscript was drafted by Dr Sehlulekile Gumede-Moyo with contributions from all the authors. All authors read and approved the final manuscript.

Availability of Data

No additional data available.

Competing interests

None declared.

Acknowledgements

The study was supported by the SEARCH (Sustainable Evaluation through Analysis of Routinely Collected HIV data) Project funded by the Bill & Melinda Gates Foundation grant number OPP1084472. We also thank the Ministry of Health, Department of Policy and Planning for granting us permission to access the SmartCare database. The authors thank all the participants who contributed their time and effort to the study and Dr. Jasleen Singh (MSc student) for going through the transcripts and assessing the initial analysis.

For peer review only

References

1. Gourlay A, Wringe A, Todd J, Michael D, Reniers G, Urassa M, et al. Challenges with routine data sources for PMTCT program monitoring in East Africa: insights from Tanzania. Global health action. 2015;8:29987.
2. Grzeskowiak LE, Gilbert AL, Morrison JL. Methodological challenges in using routinely collected health data to investigate long-term effects of medication use during pregnancy. Ther Adv Drug Saf. 2013;4(1):27-37.
3. Shaw V. Health information system reform in South Africa: developing an essential data set. . Bulletin of the World Health Organization. 2005;83(8):632–6.
4. Mate KS, Bennett B, Mphatswe W, Barker P, Rollins N. Challenges for routine health system data management in a large public program to prevent mother-to-child HIV transmission in South Africa. PloS one. 2009;4(5):e5483.
5. Munthali T. MP, Mee P., Gumede S., Schaap A, Mwinga A., Phiri C., Kapata N., Michelo C., and Todd J. Underutilisation of routinely collected data in the HIV program in Zambia: A review of quantitatively analysed peer-reviewed articles. BMC Research Policy and Systems. 2017;15(51).
6. Gumede-Moyo S, Filteau S, Munthali T, Todd J, Musonda P. Implementation effectiveness of revised (post-2010) World Health Organization guidelines on prevention of mother-to-child transmission of HIV using routinely collected data in sub-Saharan Africa: A systematic literature review. Medicine (Baltimore). 2017;96(40):e8055.
7. Russell A, Hellawell G. The future of electronic health records. Br J Hosp Med (Lond). 2013;74(11):604-5.
8. Namulanda G, Qualters J, Vaidyanathan A, Roberts E, Richardson M, Fraser A, et al. Electronic health record case studies to advance environmental public health tracking. J Biomed Inform. 2018;79:98-104.
9. Cifuentes M, Davis M, Fernald D, Gunn R, Dickinson P, Cohen DJ. Electronic Health Record Challenges, Workarounds, and Solutions Observed in Practices Integrating Behavioral Health and Primary Care. J Am Board Fam Med. 2015;28 Suppl 1:S63-72.
10. Fraser HS, Biondich P, Moodley D, Choi S, Mamlin BW, Szolovits P. Implementing electronic medical record systems in developing countries. Inform Prim Care. 2005;13(2):83-95.
11. Allen C, Jazayeri D, Miranda J, Biondich PG, Mamlin BW, Wolfe BA, et al. Experience in implementing the OpenMRS medical record system to support HIV treatment in Rwanda. Stud Health Technol Inform. 2007;129(Pt 1):382-6.
12. Siika AM, Rotich JK, Simiyu CJ, Kigotho EM, Smith FE, Sidle JE, et al. An electronic medical record system for ambulatory care of HIV-infected patients in Kenya. Int J Med Inform. 2005;74(5):345-55.
13. Oluoch T, Santas X, Kwaro D, Were M, Biondich P, Bailey C, et al. The effect of electronic medical record-based clinical decision support on HIV care in resource-constrained settings: a systematic review. Int J Med Inform. 2012;81(10):e83-92.
14. Singh J, Filteau S, Todd J, Gumede-Moyo S. Progress in the performance of HIV early infant diagnosis services in Zambia using routinely collected data from 2006 to 2016. BMC public health. 2018;18(1):1297.
15. Gumede-Moyo S, Todd J, Schaap A, Mee P, Filteau S. Effect of prevention of mother-to-child transmission strategies on antiretroviral therapy coverage in pregnant women in Zambia: analysis using routinely collected data (2010–15). Lancet Global Health. 2019;7(Supplement 1):S25.
16. Muyunda G. Zambia leads the way in SmartCare electronic health records system, a benefit to both providers and patients 2011 [Available from:

For peer review only - http://bmjopen.bmj.com/site/about/guidelines.xhtml

https://www.jhpiego.org/success-story/zambia-leads-the-way-in-smartcare-electronic-health-records-system-a-benefit-to-both-providers-and-patients/.
17. Lincoln NKDYS. Handbook of Qualitative Research 2nd ed. London: Sage Publications; 2000.
18. Vaismoradi M, Turunen H, Bondas T. Content analysis and thematic analysis: Implications for conducting a qualitative descriptive study. Nurs Health Sci. 2013;15(3):398-405.
19. Graneheim U.H. LB. Qualitative Content Analysis in Nursing Research: Concepts, Procedures and Measures to Achieve Trustworthiness. Nurse Education Today 2004;24:105-12.
20. Williams F, Boren SA. The role of the electronic medical record (EMR) in care delivery development in developing countries: a systematic review. Inform Prim Care. 2008;16(2):139-45.
21. Ivers NM, Sales A, Colquhoun H, Michie S, Foy R, Francis JJ, et al. No more 'business as usual' with audit and feedback interventions: towards an agenda for a reinvigorated intervention. Implementation science : IS. 2014;9:14.
22. Abiy R, Gashu K, Asemaw T, Mitiku M, Fekadie B, Abebaw Z, et al. A Comparison of Electronic Medical Record Data to Paper Records in Antiretroviral Therapy Clinic in Ethiopia: What is affecting the Quality of the Data? Online journal of public health informatics. 2018;10(2):e212.
23. Odekunle FF, Odekunle RO, Shankar S. Why sub-Saharan Africa lags in electronic health record adoption and possible strategies to increase its adoption in this region. International journal of health sciences. 2017;11(4):59-64.
24. Fritz F, Tilahun B, Dugas M. Success criteria for electronic medical record implementations in low-resource settings: a systematic review. J Am Med Inform Assoc. 2015;22(2):479-88.
25. Hemler JR, Hall JD, Cholan RA, Crabtree BF, Damschroder LJ, Solberg LI, et al. Practice Facilitator Strategies for Addressing Electronic Health Record Data Challenges for Quality Improvement: EvidenceNOW. J Am Board Fam Med. 2018;31(3):398-409.
26. Douglas GP, Gadabu OJ, Joukes S, Mumba S, McKay MV, Ben-Smith A, et al. Using touchscreen electronic medical record systems to support and monitor national scale-up of antiretroviral therapy in Malawi. PLoS medicine. 2010;7(8).
27. Greenhalgh T, Robert G, Macfarlane F, Bate P, Kyriakidou O. Diffusion of innovations in service organizations: systematic review and recommendations. The Milbank quarterly. 2004;82(4):581-629.

For peer review only

For peer review only - http://bmjopen.bmj.com/site/about/guidelines.xhtml

Table1: Breakdown of In-depth Interviews and Focus Group Discussions Participants

**In-depth interviews with key informants**	
Leadership	2 Implementing partners, 1 Ministry of Health representative
Database management	1 Ministry of Health ICT Officer, 2 Data Managers, 2 Monitoring and Evaluation Officers, 1 Database Developer
Data Entry Clerks	1 computer-based health facility, 4 paper-based health facilities
MCH- Nurse-in charge	1 computer –based health facility, 2 paper-based health facility
**Focus group discussions with service users**	
Peri-urban health facility	4 pregnant, 2 lactating women
Paper- based health facility	4 pregnant, 4 lactating women
Computer-based health facility	4 pregnant, 4 lactating women

For peer review only

For peer review only - http://bmjopen.bmj.com/site/about/guidelines.xhtml

Figure Legends

Figure 1

The Ministry of Health of Republic of Zambia is the copyright holder of the figure

Figure 1 Abbreviations

ANC	Antenatal Care
EHR	Electronic Health Records
HMIS	Health Management Information System
HIA	Health Information Aggregation
TB	Tuberculosis
OPD	Out Patient Department

Figure 2

The Ministry of Health of Republic of Zambia is the copyright holder of the figure

For peer review only

For peer review only - http://bmjopen.bmj.com/site/about/guidelines.xhtml

Figure 1: SmartCare Model Structure

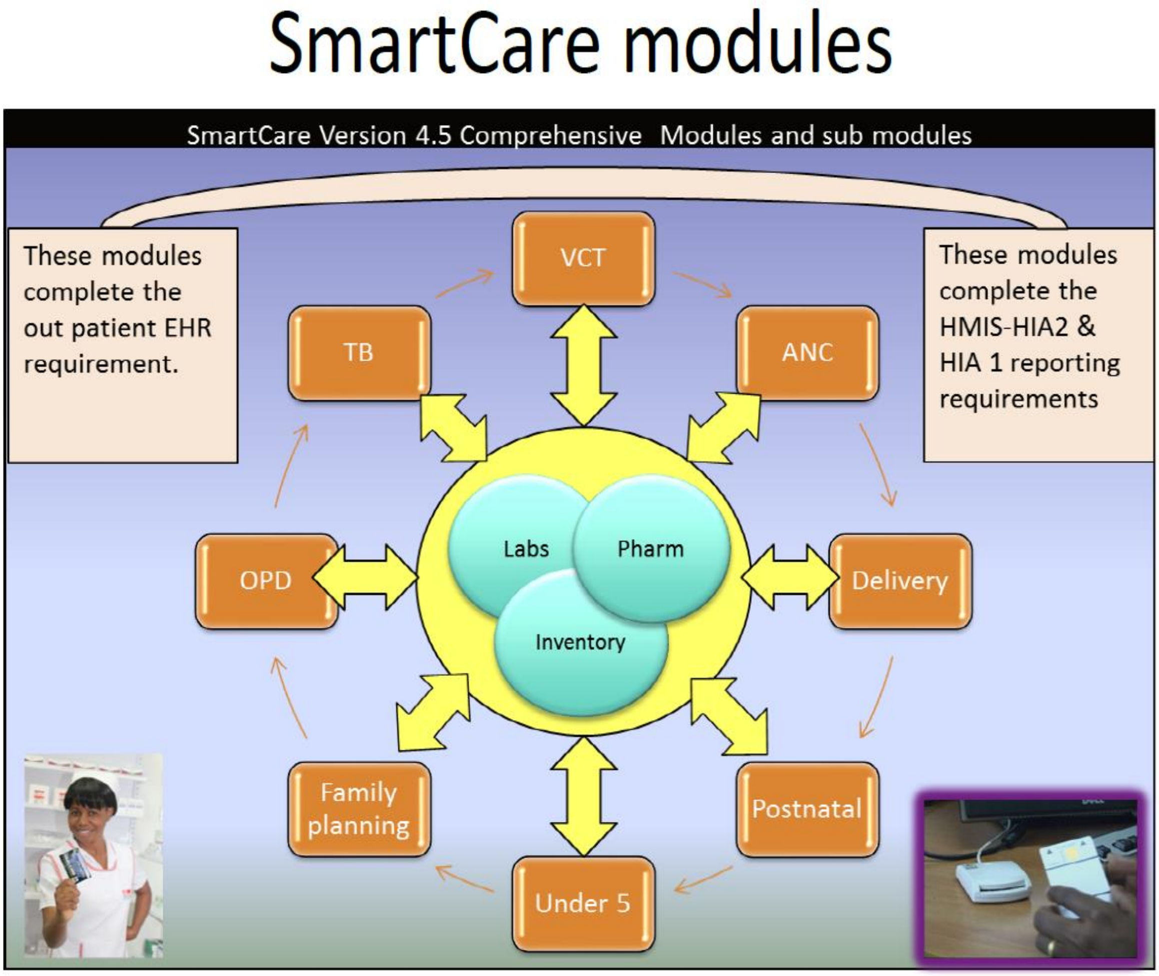

Adapted from Ministry Health HMIS workshop Presentation 2016 - ***Permission granted from the copyright holder***

w only

Figure 2: SmartCare Data flow

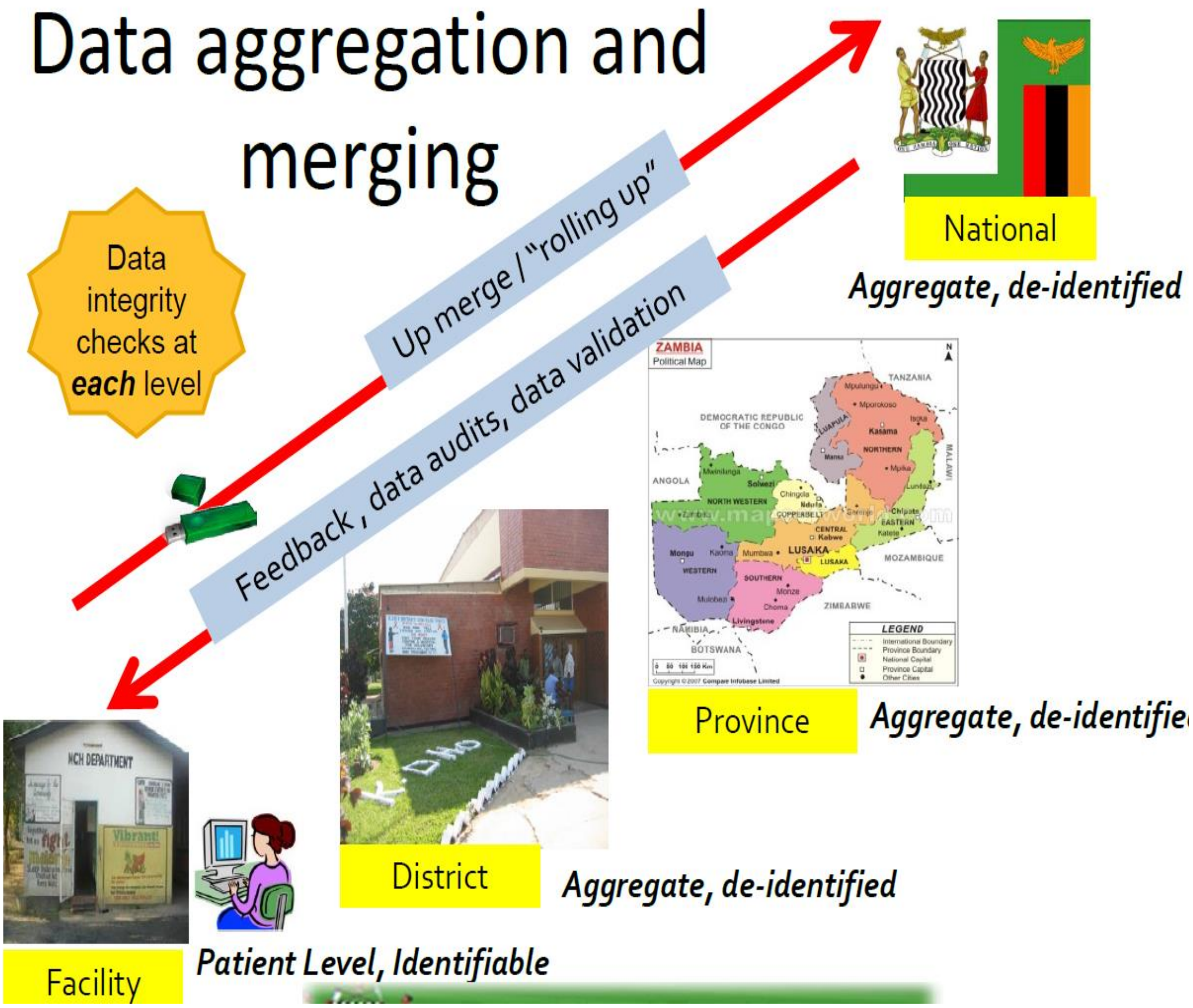

Adapted from Ministry Health HMIS workshop Presentation 2016 - ***Permission granted from the copyright holder***

COREQ (COnsolidated criteria for REporting Qualitative research) Checklist

A checklist of items that should be included in reports of qualitative research. You must report the page number in your manuscript where you consider each of the items listed in this checklist. If you have not included this information, either revise your manuscript accordingly before submitting or note N/A.

Topic	Item No.	Guide Questions/Description	Reported on Page No.
**Domain 1: Research team and reflexivity**			
Personal characteristics			
Interviewer/facilitator	1	Which author/s conducted the interview or focus group?	7
Credentials	2	What were the researcher's credentials? E.g. PhD, MD	1
Occupation	3	What was their occupation at the time of the study?	1
Gender	4	Was the researcher male or female?	N/A
Experience and training	5	What experience or training did the researcher have?	1
Relationship with participants			
Relationship established	6	Was a relationship established prior to study commencement?	4
Participant knowledge of the interviewer	7	What did the participants know about the researcher? e.g. personal goals, reasons for doing the research	6
Interviewer characteristics	8	What characteristics were reported about the inter viewer/facilitator? e.g. Bias, assumptions, reasons and interests in the research topic	6-7
**Domain 2: Study design**			
Theoretical framework			
Methodological orientation and Theory	9	What methodological orientation was stated to underpin the study? e.g. grounded theory, discourse analysis, ethnography, phenomenology, content analysis	5
Participant selection			
Sampling	10	How were participants selected? e.g. purposive, convenience, consecutive, snowball	6-7
Method of approach	11	How were participants approached? e.g. face-to-face, telephone, mail, email	7
Sample size	12	How many participants were in the study?	
Non-participation	13	How many people refused to participate or dropped out? Reasons?	6
Setting			
Setting of data collection	14	Where was the data collected? e.g. home, clinic, workplace	7
Presence of non-participants	15	Was anyone else present besides the participants and researchers?	7-8
Description of sample	16	What are the important characteristics of the sample? e.g. demographic data, date	6 & 7
Data collection			
Interview guide	17	Were questions, prompts, guides provided by the authors? Was it pilot tested?	7
Repeat interviews	18	Were repeat inter views carried out? If yes, how many?	17
Audio/visual recording	19	Did the research use audio or visual recording to collect the data?	7-8
Field notes	20	Were field notes made during and/or after the inter view or focus group?	8
Duration	21	What was the duration of the inter views or focus group?	7
Data saturation	22	Was data saturation discussed?	N/A
Transcripts returned	23	Were transcripts returned to participants for comment and/or	N/A

For peer review only

Topic	Item No.	Guide Questions/Description	Reported on Page No.
		correction?	
**Domain 3: analysis and findings**			
Data analysis			
Number of data coders	24	How many data coders coded the data?	8
Description of the coding tree	25	Did authors provide a description of the coding tree?	9
Derivation of themes	26	Were themes identified in advance or derived from the data?	8
Software	27	What software, if applicable, was used to manage the data?	8
Participant checking	28	Did participants provide feedback on the findings?	N/A
Reporting			
Quotations presented	29	Were participant quotations presented to illustrate the themes/findings? Was each quotation identified? e.g. participant number	8
Data and findings consistent	30	Was there consistency between the data presented and the findings?	N/A
Clarity of major themes	31	Were major themes clearly presented in the findings?	9-15
Clarity of minor themes	32	Is there a description of diverse cases or discussion of minor themes?	15-17

Developed from: Tong A, Sainsbury P, Craig J. Consolidated criteria for reporting qualitative research (COREQ): a 32-item checklist for interviews and focus groups. *International Journal for Quality in Health Care*. 2007. Volume 19, Number 6: pp. 349 – 357

Once you have completed this checklist, please save a copy and upload it as part of your submission. DO NOT include this checklist as part of the main manuscript document. It must be uploaded as a separate file.

For peer review only
